# Programmatic effectiveness of tuberculosis preventive treatment for people with HIV in six high-tuberculosis-burden countries (PROTECT): a prospective cohort study and meta-analysis

**DOI:** 10.1016/S2352-3018(26)00140-2

**Published:** 2026-08-17

**Authors:** N Sarita Shah, Fadimatu Mishara, Seth Zissette, Romaine Nephtalie Mesidor, Solomon Ssevvume, Serhii Riabokon, Blessing Mushangwe, John Motoku, Stephen Balogun, Nassir Kashmiri, Maryam Bello, Gerry Marcel Augustin, Deus Lukoye, Ezra J Barzilay, Talent Maphosa, Herman Weyenga, Ademola Oladipo, Odile Ferroussier-Davis, Juliana da Silva, Julia Ershova, Aderonke S Ajiboye, Brigette L Gleason, Jean Solon Valles, Kurt Jean-Charles, Thomas Achia, David Benkeser, Macarthur Charles, PROTECT study team, Reema Bhakta, Kesner François, Dianne Jean-Francois, Celestin Nicasky, Milo Richard, Jean Gregory Solide, Ibrahim Lutalo, Simon Muchuro, Proscovia Namuwenge, Simon Walusimbi, Larysa Hetman, Petro Martynov, Sherri Pals, Nataliya Podolchak, Olutomi Sodeke, Simbareshe Chaputsira, Rumbidizai Dhliwayo, Gloria Gonese, Danai Madzivire, Chiedza Marisa, Wadzanai Munakamwe, Wilson Waithaka, Samuel Abujah, Minal Ahson, Chukwuma Anyaike, Kehinde Balogun, Adebobola Bashorun, Isa Daniel, Victor Temitope Falokun, Ibrahim Bola Gobir, Francis Muoghalu, Pringar Niyang, Flora Nwagagbo, Nnamdi Umeh

**Affiliations:** a Department of Epidemiology, Rollins School of Public Health, Emory University, Atlanta, GA, USA; b Department of Medicine, School of Medicine, Emory University, Atlanta, GA, USA; c National Foundation for the Centers for Disease Control and Prevention, Atlanta, GA, USA; d Catholic Medical Mission Board, Port-au-Prince, Haiti; e Monitoring and Evaluation Technical Support Program, Makere University School of Public Health, Kampala, Uganda; f Public Health Center of Ukraine, Ministry of Health, Kyiv, Ukraine; g Zimbabwe Technical Assistance, Training & Education Center for Health, Harare, Zimbabwe; h Eastern Deanery AIDS Relief Program, Nairobi, Kenya; i Division of Global HIV and Tuberculosis, US Centers for Disease Control and Prevention Nigeria, Abuja, Nigeria; j Division of Global HIV and Tuberculosis, US Centers for Disease Control and Prevention Haiti, Port-au-Prince, Haiti; k Division of Global HIV and Tuberculosis, US Centers for Disease Control and Prevention Uganda, Kampala, Uganda; l Division of Global HIV and Tuberculosis, US Centers for Disease Control and Prevention Ukraine, Kyiv, Ukraine; m Division of Global HIV and Tuberculosis, US Centers for Disease Control and Prevention Zimbabwe, Harare, Zimbabwe; n Division of Global HIV and Tuberculosis, US Centers for Disease Control and Prevention Kenya, Nairobi, Kenya; o Division of Global HIV and Tuberculosis, US Centers for Disease Control and Prevention, Atlanta, GA, USA; p Solutions, Port-au-Prince, Haiti; q Department of Biostatistics and Bioinformatics, Rollins School of Public Health, Emory University, Atlanta, GA, USA

## Abstract

**Background:**

In randomised clinical trials (RCTs), tuberculosis preventive treatment (TPT) reduced tuberculosis incidence and mortality among people with HIV; however, real-world impact has not been evaluated since antiretroviral therapy (ART) was expanded to all people with HIV regardless of CD4 cell count. This study aimed to determine the programmatic effectiveness of TPT on reducing tuberculosis incidence and all-cause mortality among people with HIV initiating ART.

**Methods:**

This prospective cohort study emulated six RCTs using individual-level data from electronic health records in Haiti, Kenya, Nigeria, Uganda, Ukraine, and Zimbabwe to estimate TPT effectiveness among people with HIV initiating ART between Jan 1, 2018, and Dec 31, 2021 (Haiti, Kenya, Nigeria, and Ukraine) or Dec 31, 2022 (Uganda and Zimbabwe). In each country, we created cohorts of newly enrolled people with HIV initiating ART, regardless of age. We excluded people diagnosed with tuberculosis 6 months before to 30 days after ART start. De-identified extracted data for each patient included visit-level data for selected study variables between the date of enrolment into HIV care and Dec 31, 2023 (Haiti, Kenya, Nigeria, and Ukraine) or Dec 31, 2024 (Uganda and Zimbabwe). Target trial emulation was used to define a hypothetical randomised trial of TPT initiation during an 8-week grace period compared with no TPT initiation. TPT effectiveness was summarised using adjusted hazard ratios (HRs) for tuberculosis and all-cause mortality estimated using an inverse propensity weighting approach, with weights adjusted for confounders including age and sex (time-fixed) and CD4 cell count, viral load, clinical stage, ART initiation, and ART adherence (time-varying). The six countries were pooled in a meta-analysis using inverse-variance weighting with effect heterogeneity assessed using *I^2^*.

**Findings:**

We included 235 424 people with HIV: 69 936 people from Haiti, 65 936 from Uganda, 57 851 from Ukraine, 24 099 from Zimbabwe, 9549 from Kenya, and 8053 from Nigeria. In 235 424 people, 147 667 (62·7%) were aged 25–44 years, 133 582 (56·7%) were female, 101 842 (43·3%) were male, 104  277 (44·3%) had clinical stage 1, and 138 914 (59·0%) were on dolutegravir-based regimens. The adjusted HR for tuberculosis ranged from 0·35 (95% CI 0·28–0·43) in Haiti to 0·60 (0·36–0·91) in Kenya. The pooled HR for tuberculosis was 0·39 (0·34–0·43) with minimal variation across countries (*I^2^*=0%, 95% CI 0–75). The adjusted HR for mortality ranged from 0·25 (95% CI 0·20–0·30) in Uganda to 0·69 (0·53–0·96) in Zimbabwe. The pooled HR for mortality was 0·55 (0·51–0·58) with significant variation across countries (*I^2^*=96%, 95% CI 93–97).

**Interpretation:**

TPT was effective in reducing tuberculosis incidence and mortality in people with HIV across various epidemiological contexts and risk groups. In an era of universal test-and-treat and new ART regimens, these data reinforce support for TPT as essential in HIV care.

**Funding:**

Gates Foundation.

**Translations:**

For the French and Ukrainian translations of the abstract see Supplementary Materials section.

## Introduction

Tuberculosis is preventable and curable yet remains the leading infectious cause of death globally, with 10·7 million new cases and 1·2 million deaths in 2024.^1^ Among people with HIV, tuberculosis accounts for about a quarter of all AIDS-related deaths, despite substantial progress with antiretroviral therapy (ART) coverage and viral suppression over the last two decades.^2^ Preventing tuberculosis among people who have evidence of latent infection or who reside in high-tuberculosis-burden settings is a global priority, with over 25 clinical trials showing the effectiveness of tuberculosis preventive treatment (TPT) in reducing tuberculosis incidence and mortality, including among people with HIV.^3^, ^4^, ^5^, ^6^, ^7^, ^8^, ^9^, ^10^ Most of these trials were conducted early in the HIV epidemic when ART was initiated based on degree of immunosuppression (ie, CD4 cell count thresholds) and showed that TPT reduced tuberculosis incidence by 32–62%. In a trial published in 2017, people with HIV who received TPT in Cote d’Ivoire had a 46% reduction in mortality.^3^ Yet, implementation of this life-saving treatment has lagged due to concerns about drug resistance, pill burden, and overlapping toxicity with ART.^11^, ^12^ A pivotal moment occurred at the 2018 UN High Level Meeting for tuberculosis when the US President's Emergency Plan for AIDS Relief (PEPFAR) committed to providing one course of TPT to all people with HIV in care. By 2023, approximately 13 million people with HIV had received TPT through PEPFAR-supported clinical services.^13^ However, there are few data on the large-scale programmatic effectiveness of TPT, particularly in the treat all era whereby ART is recommended for all people with HIV, regardless of CD4 cell count at HIV diagnosis.

**Research in context**

**Evidence before this study**

We searched PubMed with the search terms “tuberculosis”, “preventive”, “treatment”, AND “HIV” for studies published from Jan 1, 2016, to Dec 31, 2025, without language restriction. We reviewed over 170 randomised controlled trials and over 140 systematic reviews and meta-analyses. The most recent systematic review and meta-analysis of tuberculosis preventive therapy (TPT) for tuberculosis prevention in people with HIV evaluated three randomised trials that were published between 2014 and 2017. The review found that the risks for tuberculosis and all-cause mortality were lower in participants receiving TPT with antiretroviral therapy (ART) than in participants given ART alone, but the difference in risk for all-cause mortality was non-significant. One trial was conducted in a high multidrug resistant tuberculosis burden setting. There were three observational studies, of which two evaluated treatment outcomes: one study in two primary care clinics in rural Eswatini (n=288) and one study in 14 ART centres in southern India (n=4706). The study in India followed up 4706 people with HIV for a total of 23 414 person-years and evaluated adjusted hazard for tuberculosis and mortality. They found an 87% reduction in tuberculosis and 94% reduction in all-cause mortality among people with HIV who received tuberculosis preventive treatment and ART compared with those who did not, with durable protection to 5 years.

**Added value of this study**

To the best of our knowledge, this study is the largest observational study to evaluate the impact of TPT. Furthermore, this study is the first TPT study that addresses known biases of observational data, including immortal time bias, lead time bias, and selection bias, by using target trial emulation to estimate causal effectiveness. Our study includes six nationally or regionally (within country) representative cohorts analysed as individual clinical trials and combined in a pooled meta-analysis. We expand on evidence from previous clinical trials by showing that TPT has protective benefit in real-world settings across diverse epidemiological contexts, even among people with HIV on ART who are virologically suppressed.

**Implications of all the available evidence**

In an era of universal test-and-treat and new ART regimens, these data reinforce support for TPT as essential in HIV care. Our analytic approach and sensitivity analysis allowed for variation in the grace period for TPT initiation based on programme realities or requirements and found TPT was most beneficial when started early. Although this study evaluated a 6-month isoniazid regimen, future research needs to assess the impact of new, short-course TPT on tuberculosis incidence and mortality while simultaneously targeting the structural barriers that delay TPT initiation within real-world clinics. Given current global funding challenges, ensuring continued vigilance for TPT delivery to people with HIV will be critical to ensure gains are sustained.

In addition to TPT scale-up, developing and strengthening health information systems has been a priority for PEPFAR, which has supported countries to implement electronic medical records (EMRs) for clinical management of people with HIV over the past decade.^14^, ^15^ These systems offer opportunities to integrate patient care across disease programmes (eg, HIV, tuberculosis, and COVID-19), and to evaluate programme performance and the effect of interventions.^16^ However, use of routine clinical data to answer questions about the causal effectiveness of interventions can be challenging due to known biases of observational data, such as immortal time bias, lead time bias, and selection bias.^17^ Nonetheless, observational data offer unique opportunities to evaluate questions that might not be feasibly or ethically addressed with randomised trials. To minimise biases of observational data, a target trial framework can be used to emulate a clinical trial by clearly defining parameters of a hypothetical trial (eg, study eligibility, treatment assignment, follow-up time) and aligning the observational data to these criteria.^18^ Target trial emulation has been used to examine treatment interventions across a wide array of medical conditions, primarily using EMR or claims data;^19^ however, use in tuberculosis research has been limited.^19^, ^20^, ^21^

The Preventing Occurrence of Tuberculosis by Expanding Coverage of TPT Among People with HIV (PROTECT) study was designed to examine the real-world effectiveness of TPT among population-based cohorts in six high-tuberculosis-burden countries: Haiti, Kenya, Nigeria, Uganda, Ukraine, and Zimbabwe. In 2024, total tuberculosis incidence in these countries ranged from 86 cases per 100 000 people (Ukraine) to 207 cases per 100 000 people (Kenya; appendix 3 pp 4–5).^1^ TPT was scaled-up nationwide earlier in Kenya (2015) and Haiti (2017), while the remaining countries accelerated scale-up from 2018 onwards. Universal ART (ie, treat all) for people with HIV was fully implemented by 2018 in all countries in this study. Short TPT regimens, such as 3-month isoniazid and rifapentine, are known to improve treatment completion and were being piloted in some countries in 2020 but were not widely available for people with HIV during the study period. We applied a target trial framework to observational data obtained from EMRs and surveillance data to determine the programmatic effectiveness of TPT on reducing tuberculosis incidence and all-cause mortality among people with HIV initiating ART.

## Methods

### Study design and participants

This prospective cohort study used target trial emulation to analyse individual-level data from electronic medical records in Haiti, Kenya, Nigeria, Uganda, Ukraine, and Zimbabwe of people with HIV initiating ART in PEPFAR-supported clinical sites. No additional clinical training or intervention was provided as part of this study. HIV care, tuberculosis screening and treatment, and TPT initiation followed each country's national guidelines and remained at providers’ discretion. The tuberculosis and HIV burden and guidelines for tuberculosis diagnosis and TPT provision to people with HIV in the six countries are detailed in the appendix 3 (pp 4–5).

Each country developed its own study protocol, which was reviewed and approved by ethics committees in that country: Haiti National Bioethics Committee (2223–6), Kenyatta National Hospital Ethical Review Committee (SU-ISERC1510/22), Nigeria's National Health Research Ethics Committee (NHREC/01/01/2007–22/07/2022), Uganda's Makerere University School of Public Health (also registered with the Uganda National Council for Science and Technology), Public Health Center of the Ministry of Health in Ukraine (Number 102), and Medical Research Council of Zimbabwe (MRCZ/E/315). The protocols were reviewed by the US Centers for Disease Control and Prevention (CDC) and each was deemed not to be research and were conducted consistent with applicable federal law and CDC policy. There was a statistical analysis plan that was applied to all countries for each trial emulation.^22^ Informed consent was not obtained because the study used routinely collected, de-identified data and involved no interaction with patients. The evaluation posed no more than minimal risk and did not affect the rights, welfare, or care received by patients. Given the scale of the dataset and the inability to feasibly contact all individuals whose records were included, the study could not practicably be conducted without a waiver of consent.

### Procedures

In Haiti and Ukraine, data were extracted for all people with HIV in care nationwide. For Haiti, the study used data from the Suivi Actif Longitudinal du VIH en Haïti system, a case-based de-duplicated longitudinal database capturing all individuals receiving HIV care nationwide, which includes 165 PEPFAR-supported health facilities.^23^ Tuberculosis diagnosis and treatment information was sourced from the national tuberculosis tracker and merged with the HIV data. For Ukraine, details of the HIV and tuberculosis surveillance systems, and the process for merging datasets have been previously reported.^24^ Briefly, we crossmatched data from eTB Manager, the national tuberculosis electronic database, with records in the HIV Medical Information System, a national EMR used by HIV treatment sites nationwide, using multiple identifiers to verify tuberculosis diagnosis among people with HIV.

In Uganda, Zimbabwe, and Nigeria the study population was selected based on a combination of national representativeness, feasibility of accessing patient charts for data quality assessments, and functionality of the EMR at sites. For Uganda, 54 facilities were selected to reflect the distribution of people with HIV in care and to include a mix of facility levels (primary, secondary, and tertiary) and ownership types (public *vs* private not-for-profit). All patients who met eligibility criteria at the participating sites were included. Data were extracted from sites’ UgandaEMR or ClinicMaster systems.

For Zimbabwe, a cluster-based approach was used to calculate the sample size needed to provide national estimates, with health facilities as primary sampling units (or clusters). 15 facilities from six provinces were selected where the EMR system was well established in order to reach the minimum required sample size of 20 000 people with HIV. For Nigeria, 60 facilities across nine states were selected using multi-stage probability sampling, targeting a sample size needed of 18 397. Additional considerations included human and financial resources, safety, and disruptions caused by the COVID-19 pandemic.

Regional data were extracted from the Ecare EMR for all people with HIV in Kenya who were receiving care at sites supported by the Eastern Deanery AIDS Relief Program, a PEPFAR implementing partner serving 29 000 people with HIV across 14 sites in one county in eastern Nairobi.

Across all six countries, de-identified extracted data for each patient included visit-level data for selected study variables between the date of enrolment into HIV care and Dec 31, 2023 (Haiti, Kenya, Nigeria, and Ukraine) or Dec 31, 2024 (Uganda and Zimbabwe). At each site, paper-based HIV and tuberculosis registers and patients’ clinical files were compared with EMR data to ensure completeness and consistency for key variables (appendix 3 pp 6–7). All datasets were thoroughly assessed for quality issues, including missing data and date inconsistencies, and cleaned iteratively. A missing category was coded when data were missing for categorical variables, while a separate indicator of missingness was created for numeric variables and used to identify when values were measured over time. Contact with a person known to have tuberculosis or drug-resistant tuberculosis was not available as a covariate.

Data on sex were retrieved from the EMRs. Data on race and ethnicity were not available in EMRs due to local data governance policies and the sensitive nature of these identifiers for people with HIV.

### Outcomes

The primary outcome was the incidence of laboratory-confirmed or clinically diagnosed tuberculosis. A participant was considered to have had a tuberculosis episode if their record included at least one of the following variables: tuberculosis diagnosis date, tuberculosis treatment start date, bacteriological test result with *Mycobacterium tuberculosis* detected, or anti-tuberculosis treatment prescription (ie, four-drug tuberculosis disease treatment regimen). A coprimary outcome was all-cause mortality, as defined by a vital status recorded as died and a date of death. Those with missing date of death were right censored for death based on the date of their last clinic visit.

### Statistical analysis

A clinical trial was emulated by specifying parameters of a target trial for a realistic TPT programme. The study cohort was defined as all newly enrolled people with HIV initiating ART between Jan 1, 2018, up to Dec 31, 2021 (Haiti, Kenya, Nigeria, and Ukraine) or Dec 31, 2022 (Uganda and Zimbabwe) at selected sites or nationwide. People transferring care from other clinical sites and people with recent tuberculosis diagnosis (tuberculosis disease 180 days before to 30 days after ART initiation) were excluded. The intervention of interest was defined as initiating a first course of TPT within 8 weeks of enrolling into HIV care (ie, the grace period for target trial) to reflect real-world programmatic realities whereby patients could be undergoing tuberculosis diagnostic evaluation, if symptomatic, or facilities could face stock-outs or other factors that might delay same-day initiation of TPT. This intervention was compared with the control condition of never receiving TPT. Follow-up time began on the date of initial enrolment in HIV care and lasted until tuberculosis diagnosis, death, right censoring, or administrative censoring after 2 years, in line with time periods used for follow-up in previous clinical trials. Covariates of interest varied by country (appendix 3 p 8) but generally included time-fixed covariates such as enrolment year, age and sex, as well as time-varying covariates including CD4 cell count, viral load, WHO clinical stage, ART adherence, and tuberculosis symptoms.

Full details of our statistical analysis plan, in line with TARGET guidance, have been described elsewhere.^22^ Briefly, we used a clone-censor-weight approach that involved estimation of propensity models describing the time-varying probability of TPT initiation, and the time-varying probability of right censoring. Propensities were estimated using logistic regression and adjusted for covariates. We truncated at the 99th percentile to reduce the influence of extreme weights, in line with recommendations for inverse propensity-weighted analyses,^25^ and we examined time-varying means of the inverse weights to evaluate evidence for propensity model mis-specification.^26^ To allow for the 8-week grace period wherein patients can contribute follow-up time to both the hypothetical care programme and the control conditions, patients were cloned and inverse-weighted according to their time-varying probabilities of initiating TPT and of being right censored (appendix 3 p 9) to estimate a hazard ratio (HR) based on a pooled logistic regression marginal structural model. For the primary tuberculosis outcome, death was considered a competing event in the marginal structural model and effectiveness was summarised using a cause-specific HR.

Sensitivity analyses were conducted to characterise the effectiveness of TPT initiation over shorter or longer grace periods (2 weeks to 24 weeks, with shorter periods reflecting an ideal TPT programme and longer periods reflecting programmatic realities that might cause delays), compared with the 8 weeks selected for the primary analysis. We also varied the length of the early tuberculosis diagnosis exclusion period.

Within each country, we evaluated heterogeneity of treatment effectiveness by age (younger than 15 years, aged 15–44 years, and older than 44 years), sex, baseline ART regimen (dolutegravir-based, efavirenz-based, or other), baseline WHO clinical stage, and calendar year of enrolment into HIV care. To ensure stability of results across countries, a country-specific minimum number of cases of each endpoint (at least six incident tuberculosis cases, at least six deaths) were required to characterise effectiveness against an endpoint in a particular subgroup. Subgroups falling below this threshold were excluded from reporting due to lack of precision. We controlled for multiple testing within each country using a Bonferroni correction: the p value threshold for declaring the presence of effect modification by a particular factor in a particular country was set to 0·01.

A pooled meta-analysis was conducted to characterise the overall effectiveness of TPT in reducing tuberculosis incidence and all-cause mortality across all six countries. Pooled HRs were computed using inverse variance weighting. Heterogeneity of the effectiveness across countries was assessed using *I^2^* which estimates the proportion of variability in effectiveness results that is explained by differences between countries, rather than by sampling error.^27^ All analyses were done using R, version 4.4.0; all analysis code is available online.

Nigeria did not meet the protocol-specified target sample size so was underpowered for country-specific and subgroup analysis, but data were retained in the pooled meta-analysis. Nigeria-specific estimates are presented for completeness but were largely not estimable.

### Role of the funding source

The study sponsor provided technical review and input on the study design but did not have a role in data collection, data analysis, or data interpretation or writing of the report.

## Results

After applying eligibility criteria for the target trial, our total study population included 235 424 people with HIV, 69 936 people from Haiti, 65 936 from Uganda, 57 851 from Ukraine, 24 099 from Zimbabwe, 9549 from Kenya, and 8053 from Nigeria (appendix 3 pp 10–12). Tuberculosis diagnosed within 30 days of entering HIV care accounted for exclusion of 1685 (2·4%) of 71 621 people with HIV in Haiti, 652 (3·6%) of 18 006 people in Kenya, 18 (0·1%) of 17 396 people in Nigeria, 1331 (2·0%) of 67 267 people in Uganda, 801 (0·5%) of 156 912 people in Ukraine, and 413 (1·6%) of 25 896 people in Zimbabwe.

In 235 424 people, 147 667 (62·7%) were aged 25–44 years, 133 582 (56·7%) were female, 101 842 (43·3%) were male, 104  277 (44·3%) had clinical stage 1, and 138 914 (59·0%) were on dolutegravir-based regimens. Children with HIV younger than 15 years comprised 3·2% of the total study population, with smaller proportions in Nigeria and Ukraine (table 1; appendix 3 pp 13–24). Year of entry into care was uniformly distributed for Haiti and Ukraine but varied for the other countries. At entry into care, 44·3% of people with HIV were classified as WHO stage 1. In all countries except Ukraine, baseline CD4 cell counts were unavailable due to policy shifts that deprioritised CD4 testing at entry into care. In Ukraine, nearly a third (32%) of people entered care with CD4 counts less than 200 cells per μL, consistent with advanced HIV disease. First-measured viral load was less than 1000 copies per mL (ie, suppressed) in the majority of people with HIV in Haiti, Kenya, Nigeria, Uganda, and Zimbabwe, where the first viral load was typically measured after 6 months on ART (table 1). In Ukraine, viral load was generally measured at enrolment into HIV care. A majority of people with HIV in each country were initiated on a dolutegravir-based ART regimen, except in Uganda (42% initiated on dolutegravir-based regimen and 49% on efavirenz-based regimen) and Zimbabwe (49% dolutegravir and 38% efavirenz). TPT was initiated in the majority of people with HIV, although there was variation across countries in the proportion who initiated within the 8-week grace period, ranging from 11% in Uganda to 69% in Haiti.

**Table 1:** Demographic and clinical characteristics of study participants at entry into HIV care, by country

			**Haiti (n=69 936)**	**Kenya (n=9549)**	**Nigeria (n=8053)**	**Uganda (n=65 936)**	**Ukraine (n=57 851)**	**Zimbabwe (n=24 099)**
Age								
	<5 years		1712 (2·4%)	162 (1·7%)	56 (0·7%)	1714 (2·6%)	253 (0·4%)	421 (1·7%)
	5–14 years		1116 (1·6%)	225 (2·4%)	69 (0·9%)	1194 (1·8%)	184 (0·3%)	468 (1·9%)
	15–24 years		9882 (14·1%)	1511 (15·8%)	1997 (24·8%)	15 920 (24·1%)	2748 (4·8%)	4754 (19·7%)
	25–34 years		22 427 (32·1%)	3879 (40·6%)	3753 (46·6%)	25 288 (38·4%)	17 276 (29·9%)	8245 (34·2%)
	35–44 years		19 008 (27·2%)	2460 (25·8%)	1599 (19·9%)	13 749 (20·9%)	23 572 (40·7%)	6411 (26·6%)
	45–54 years		9889 (14·1%)	1018 (10·7%)	424 (5·3%)	5651 (8·6%)	10 139 (17·5%)	2741 (11·4%)
	>55 years		5902 (8·4%)	294 (3·1%)	155 (1·9%)	2420 (3·7%)	3679 (6·4%)	1059 (4·4%)
Sex								
	Male		27 379 (39·1%)	2994 (31·4%)	4001 (49·7%)	23 410 (35·5%)	35 437 (61·3%)	8621 (35·8%)
	Female		42 557 (60·9%)	6555 (68·6%)	4052 (50·3%)	42 526 (64·5%)	22 414 (38·7%)	15 478 (64·2%)
		Pregnant*	3954/42 557 (9·3%)	1057/6555 (16·1%)	61/4052 (1·5%)	6279/42 526 (14·8%)	-	656/15 478 (4·2%)
		Breastfeeding*†‡	..	..	22/4052 (0·5%)	561/42 526 (1·3%)	..	537/15 478 (3·5%)
Enrolment year								
	2018		18 943 (27·1%)	2171 (22·7%)	657 (8·2%)	21 512 (32·6%)	13 945 (24·1%)	3277 (13·6%)
	2019		19 334 (27·6%)	3127 (32·7%)	872 (10·8%)	15 595 (23·7%)	14 742 (25·5%)	7555 (31·3%)
	2020		17 752 (25·4%)	2532 (26·5%)	3921 (48·7%)	11 219 (17·0%)	14 889 (25·7%)	4780 (19·8%)
	2021		13 907 (19·9%)	1719 (18·0%)	2603 (32·3%)	9332 (14·2%)	14 275 (24·7%)	4412 (18·3%)
	2022§		..	..	..	8278 (12·6%)	..	4075 (16·9%)
Clinical stage¶‖								
	Stage 1		35 665/56 932 (62·6%)	4846 (50·7%)	6791/7099 (95·7%)	43 829/56 738 (77·2%)	..	13 146/20 972 (62·7%)
	Stage 2		13 329/56 932 (23·4%)	3246 (34·0%)	228/7099 (3·2%)	9595/56 738 (16·9%)	..	5092/20 972 (24·3%)
	Stage 3		6502/56 932 (11·4%)	1342 (14·1%)	69/7099 (1·0%)	2539/56 738 (4·5%)	..	2558/20 972 (12·2%)
	Stage 4		1436/56 932 (2·5%)	115 (1·2%)	11/7099 (0·2%)	775/56 738 (1·4%)	..	176/20 972 (0·8%)
	Missing		13 004 (18·6%)	0	954 (11·8%)	9198 (13·9%)	57 851 (100·0%)	3127 (13·0%)
CD4 count (cells per mm^3^)†¶**								
	0–199		..	1431/6996 (20·5%)	92/297 (31·0%)	4754/20 281 (23·4%)	17 325/53 818 (32·2%)	206/629 (32·8%)
	200–349		..	1292/6996 (18·5%)	70/297 (23·6%)	3893/20 281 (19·2%)	12 265/53 818 (22·8%)	154/629 (24·5%)
	350–499		..	1284/6996 (18·4%)	52/297 (17·5%)	3762/20 281 (18·5%)	10 182/53 818 (18·9%)	103/629 (16·4%)
	>500		..	2989/6996 (42·7%)	83/297 (27·9%)	7872/20 281 (38·8%)	14 046/53 818 (26·1%)	166/629 (26·4%)
	Missing		69 936 (100%)	2553 (26·7%)	7756 (96·3%)	45 655 (69·2%)	4033 (7·0%)	23 470 (97·4%)
Viral load (copies per mL)¶**								
	<1000		36 568/47 870 (76·4%)	5920/7295 (81·2%)	2414/2586 (93·3%)	35 909/39 091 (91·9%)	15 957/50 268 (31·7%)	13 393/14 203 (94·3%)
	1000–99 999		9835/47 870 (20·5%)	970/7295 (13·3%)	111/2586 (4·3%)	2484/39 091 (6·4%)	21 588/50 268 (42·9%)	674/14 203 (4·7%)
	≥100 000		1467/47 870 (3·1%)	405/7295 (5·6%)	61/2586 (2·4%)	698/39 091 (1·8%)	12 723/50 268 (25·3%)	136/14 203 (1·0%)
	Missing		22 066 (31·6%)	2254 (23·6%)	5467 (67·9%)	26 845 (40·7%)	7583 (13·1%)	9896 (41·1%)
ART regimen¶								
	Dolutegravir-based		53 857/62 016 (86·8%)	7004/9094 (77·0%)	6740/7490 (90·0%)	26 942/64 632 (41·7%)	32 633 (56·4%)	11 738 (48·7%)
	Efavirenz-based		6255/62 016 (10·1%)	1756/9094 (19·3%)	563/7490 (7·5%)	31 497/64 632 (48·7%)	20 009 (34·6%)	9070 (37·6%)
	Nevirapine-based		170/62 016 (0·3%)	23/9094 (0·3%)	139/7490 (1·9%)	756/64 632 (1·2%)	193 (0·3%)	160 (0·7%)
	Atazanavir–ritonavir or lopinavir–ritonavir-based		660/62 016 (1·1%)	311/9094 (3·4%)	42/7490 (0·6%)	1270/64 632 (2·0%)	4885 (8·4%)	398 (1·7%)
	Other		1074/62 016 (1·7%)	0	6/7490 (0·1%)	4167/64 632 (6·4%)	93 (0·2%)	2733 (11·3%)
	Missing		7920 (11·3%)	455 (4·8%)	563 (7·0%)	1304 (2·0%)	38 (0·1%)	0
TPT initiation relative to enrolment in HIV care								
	≤8 weeks		48 175 (68·9%)	6363 (66·6%)	3677 (45·7%)	7174 (10·9%)	25 650 (44·3%)	6214 (25·8%)
	>8 weeks		8522 (12·2%)	1397 (14·6%)	3909 (48·5%)	29 475 (44·7%)	8122 (14·0%)	9054 (37·6%)
	Never		13 239 (18·9%)	1789 (18·7%)	467 (5·8%)	29 287 (44·4%)	24 079 (41·6%)	8831 (36·6%)
Tuberculosis during follow-up			851 (1·2%)	178 (1·9%)	19 (0·2%)	775 (1·2%)	1741 (3·0%)	235 (1·0%)
Mortality during follow-up			2854 (4·1%)	324 (3·4%)	28 (0·3%)	2333 (3·5%)	3607 (6·2%)	386 (1·6%)
Follow-up time right-censored for tuberculosis††			..	4279 (44·8%)	1646 (20·4%)	33 671 (51·1%)	11 315 (19·6%)	9668 (40·1%)
Time from enrolment to tuberculosis follow-up time right-censoring (days)			..	133 (25–339)	265 (80–592)	143 (14–412)	287 (165, 453)	241 (31–505)
Follow-up time right-censored for death			33 464 (47·8%)	3167 (33·2%)	1925 (23·9%)	31 654 (48·0%)	11 015 (19·0%)	9139 (37·9%)
Time from enrolment to death follow-up time right-censoring (days)			303 (66–525)	144 (28–365)	339 (87–617)	118 (0–406)	287 (166–454)	273 (71–512)

Data are n (%), n/N (%), or median (IQR). ART=antiretroviral therapy. TPT=tuberculosis preventive treatment.

* Percent among participants who are female only.

† Not reported in Haiti data.

‡ Not reported in Kenya data.

§ 2022 data were not extracted for Haiti, Kenya, Nigeria, or Ukraine.

¶ Percentage among participants with those measurements.

‖ Not reported in Ukraine data.

** First recorded measurement, not necessarily at entry into care.

†† Tuberculosis data in Haiti captured with country-wide mandatory reporting system, indicating no missed cases.

Follow-up time contributing to each of the two outcomes varied by country (appendix 3 p 25). Estimated 2-year cumulative incidence of tuberculosis in the TPT group compared with the no TPT group is shown in figure 1A. Among countries that met the protocol-specified target sample size, adjusted HRs for tuberculosis ranged from 0·35 (95% CI 0·28–0·43) in Haiti to 0·60 (0·36–0·91) in Kenya (table 2). In Nigeria, point estimates of effectiveness were protective, but highly imprecise (adjusted HR 0·61, 95% CI 0·00–3·02) owing to the reduced sample size. The pooled HR for tuberculosis across all six countries was 0·39 (95 CI% 0·34–0·43), indicating an average reduction of 61% in tuberculosis incidence associated with receipt of TPT within the 8-week grace period compared with never receiving TPT (figure 2A). The estimated *I^2^* across the six countries was 0% (95% CI 0–75), indicating little evidence of heterogeneity of effectiveness across the countries.^28^

**Figure 1:**
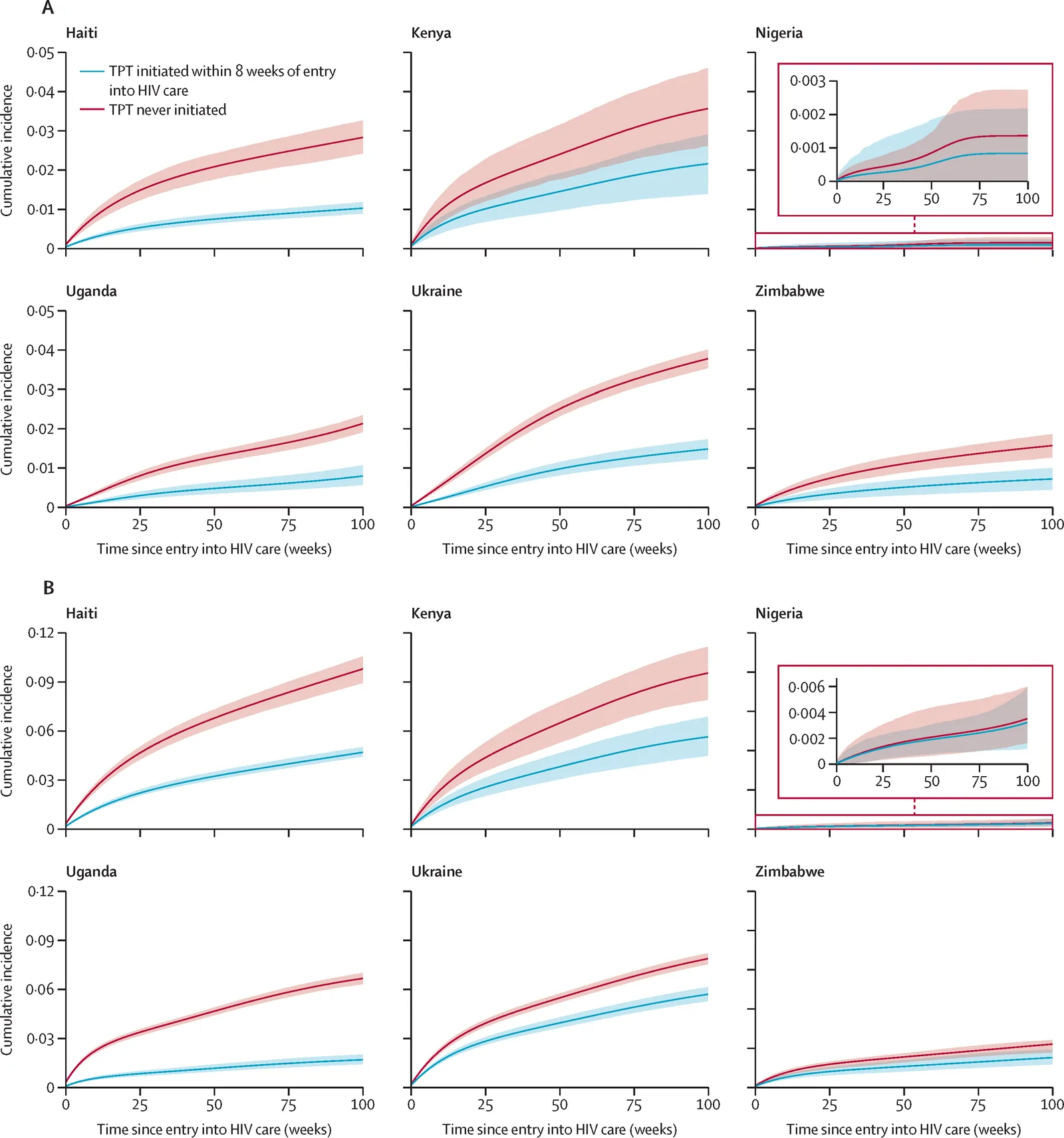
2-year cumulative incidence of tuberculosis diagnosis and all-cause mortality comparing TPT initiation within 8 weeks of enrolment into care with never initiating TPT, by country (A) Tuberculosis diagnosis. (B) All-cause mortality. Shaded areas represent 95% CI. TPT=tuberculosis preventive treatment.

**Table 2:** Adjusted hazard ratios for tuberculosis diagnosis comparing TPT initiation within 8 weeks of enrolment into care to never initiating TPT, pooled and by country

		**Pooled**	**Haiti**	**Kenya**	Nigeria*	**Uganda**	**Ukraine**	**Zimbabwe**
Overall		0·39 (0·34–0·43)	0·35 (0·28–0·43)	0·60 (0·36–0·91)	0·61 (0·00–3·02)	0·37 (0·26–0·51)	0·38 (0·32–0·45)	0·46 (0·28–0·67)
	*I^2^*	0% (0–75)	..	..	..	..	..	..
Age†								
	<15 years	0·41 (0·21–0·81)	0·40 (0·16–0·86)	1·16 (0·00–7·35)	..	0·39 (0·07–0·97)	0·66 (0·00–3·57)	0·94 (0·00–4·42)
	15–44 years	0·36 (0·32–0·42)	0·33 (0·26–0·44)	0·46 (0·25–0·76)	..	0·37 (0·24–0·53)	0·36 (0·29–0·44)	0·51 (0·28–0·81)
	>44 years	0·46 (0·36–0·58)	0·42 (0·27–0·67)	1·17 (0·39–2·94)	..	0·43 (0·14–0·81)	0·47 (0·32–0·62)	0·27 (0·10–0·56)
	p value	..	0·64	0·26	..	0·96	0·42	0·50
Sex								
	Female	0·41 (0·34–0·48)	0·32 (0·24–0·44)	0·59 (0·32–1·01)	1·58 (0·00–>10·00)	0·41 (0·25–0·65)	0·44 (0·32–0·56)	0·51 (0·22–1·00)
	Male	0·37 (0·32–0·44)	0·37 (0·28–0·50)	0·59 (0·27–1·08)	0·31 (0·00–2·78)	0·34 (0·20–0·52)	0·36 (0·29–0·45)	0·43 (0·21–0·74)
	p value	..	0·52	0·99	1·00	0·55	0·30	0·74
Enrolment year†‡								
	2018	0·42 (0·34–0·52)	0·44 (0·32–0·62)	0·51 (0·18–1·60)	..	0·34 (0·06–0·73)	0·41 (0·30–0·54)	0·54 (0·09–1·33)
	2019	0·38 (0·31–0·47)	0·31 (0·21–0·47)	0·71 (0·27–1·52)	..	0·28 (0·13–0·50)	0·41 (0·30–0·52)	0·57 (0·03–1·65)
	2020	0·37 (0·30–0·47)	0·35 (0·22–0·55)	0·20 (0·09–0·43)	..	0·42 (0·19–0·76)	0·42 (0·30–0·58)	0·43 (0·07–1·06)
	2021	0·41 (0·29–0·56)	0·25 (0·15–0·42)	1·90 (0·60–4·25)	..	0·42 (0·19–0·81)	0·30 (0·09–0·67)	0·48 (0·21–0·89)
	2022	0·34 (0·18–0·64)	..	..	..	0·56 (0·20–1·11)	..	0·18 (0·06–0·41)
	p value	..	0·33	0·0055	..	0·79	0·94	0·59
Clinical stage†§^¶^								
	Stage 1	0·57 (0·42–0·77)	0·85 (0·46–1·40)	0·61 (0·23–1·62)	..	0·43 (0·26–0·64)	..	0·61 (0·21–1·30)
	Stage 2	0·44 (0·32–0·60)	0·35 (0·19–0·44)	0·42 (0·16–0·89)	..	0·43 (0·16–0·82)	..	1·26 (0·49–2·91)
	Stage 3	0·42 (0·30–0·58)	0·33 (0·24–0·54)	0·61 (0·29–1·22)	..	0·34 (0·02–0·89)	..	0·61 (0·25–1·17)
	Stage 4	0·28 (0·19–0·39)	0·28 (0·23–0·47)	..	..	0·02 (0·00–0·07)	..	..
	p value	..	0·026	0·77	..	0·81	..	0·41
Antiretroviral therapy regimen†‖								
	Dolutegravir-based	0·39 (0·33–0·45)	0·30 (0·23–0·38)	0·58 (0·32–0·97)	..	0·42 (0·27–0·62)	0·44 (0·34–0·57)	0·43 (0·24–0·73)
	Efavirenz-based	0·36 (0·29–0·45)	0·60 (0·29–1·26)	0·21 (0·08–0·65)	..	0·31 (0·12–0·57)	0·35 (0·27–0·44)	0·82 (0·14–1·89)
	Other	0·27 (0·19–0·37)	0·12 (0·38–0·97)	..	..	0·44 (0·15–0·86)	0·76 (0·38–1·26)	0·25 (0·03–0·64)
	p value	..	0·079	0·095	..	0·77	0·035	0·49

Data are HR (95% CI), unless otherwise stated. The p value evaluates whether there is evidence that the preventive effectiveness of TPT is different by the measured characteristic. TPT=tuberculosis preventive treatment.

* Did not meet protocol-specified sample size. Results included for completeness.

† Insufficient cases to estimate by subgroup for Nigeria.

‡ 2022 data were not extracted for Haiti, Kenya, Nigeria, or Ukraine. ‡Not reported in Ukraine data.

§ Insufficient cases to estimate stage 4 subgroup for Kenya and Zimbabwe.

‖ Insufficient cases to estimate the other subgroup for Kenya.

**Figure 2:**
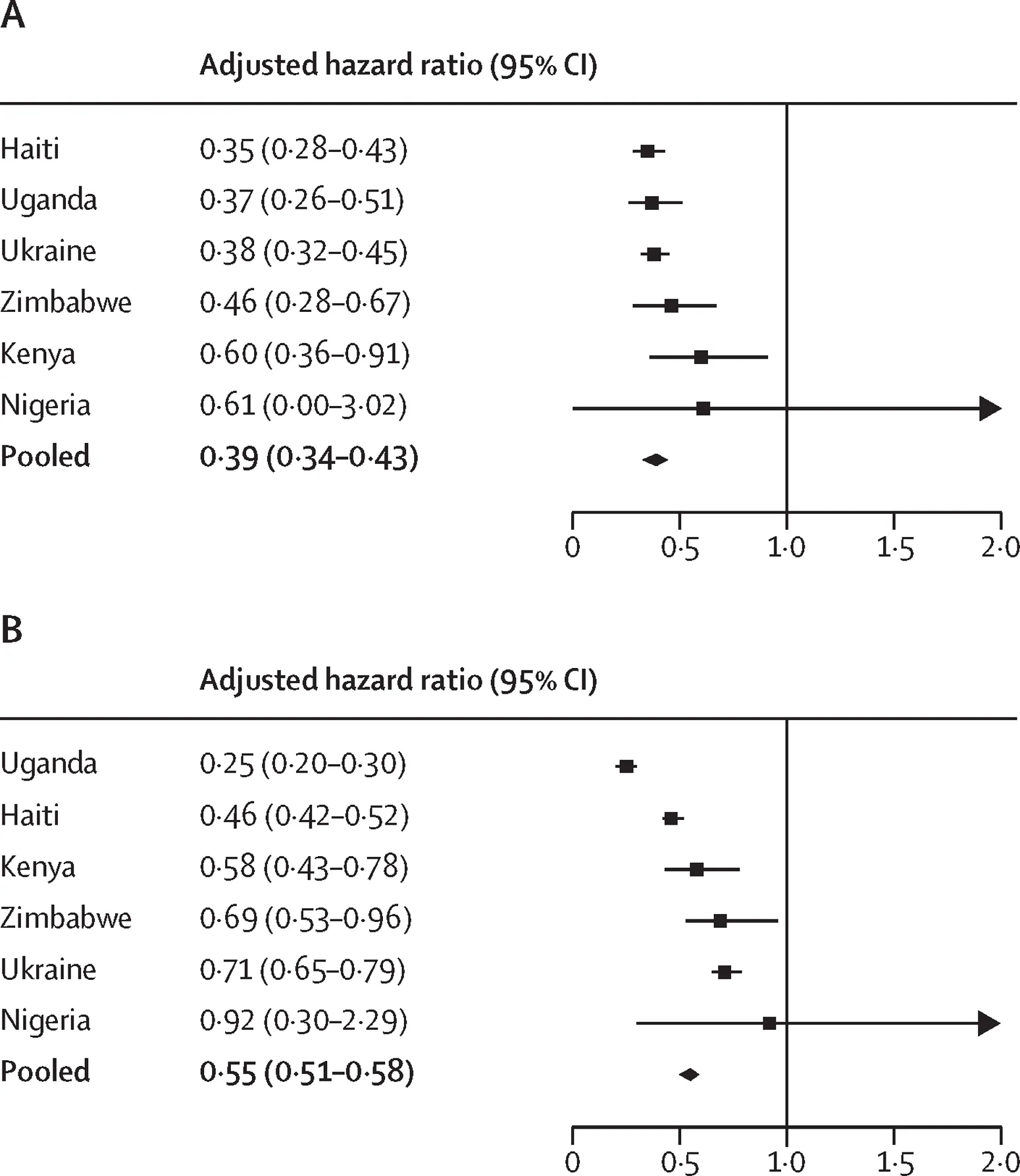
Adjusted hazard ratios of tuberculosis diagnosis and all-cause mortality comparing TPT initiation within 8 weeks of enrolment into care with never initiating TPT, pooled and by country (A) Tuberculosis diagnosis. (B) All-cause mortality. TPT=tuberculosis preventive treatment.

Estimated 2-year cumulative incidence of mortality with TPT and without TPT is shown in figure 1B. Country-specific adjusted HRs for mortality among countries that met the target sample size ranged from 0·25 (95% CI 0·20–0·30) in Uganda to 0·69 (0·53–0·96) in Zimbabwe. In pooled meta-analysis across all six countries, the overall HR was 0·55 (0·51–0·58), indicating a 45% reduction in mortality associated with receiving TPT within the 8-week grace period compared with never receiving TPT (figure 2B). Although all countries had protective estimates of effectiveness of TPT, the magnitude of that effectiveness varied considerably across countries (*I^2^*=96%, 95 CI% 93–97).

There was little evidence that effectiveness of TPT in preventing tuberculosis varied by individual characteristics of people with HIV in each country (table 2). The limited sample size in Nigeria limited the ability to draw any meaningful country-specific conclusions in these analyses, although results are displayed for completeness. After multiplicity correction, the only country to show evidence of effect heterogeneity was Kenya, where effectiveness of TPT differed significantly by enrolment year (p=0·0055). There was some evidence that effectiveness of TPT in preventing all-cause mortality varied by enrolment year in Uganda (p<0·0001) and Ukraine (p<0·0001) and by ART regimen in Haiti (p=0·0012), with stronger protective effects shown for dolutegravir-based regimens compared with efavirenz-based regimens (table 3).

**Table 3:** Adjusted hazard ratios for all-cause mortality comparing TPT initiation within 8 weeks of enrolment into care to never initiating TPT, pooled and by country

		**Pooled**	**Haiti**	**Kenya**	Nigeria*	**Uganda**	**Ukraine**	**Zimbabwe**
Overall		0·55 (0·51–0·58)	0·46 (0·42–0·52)	0·58 (0·43–0·78)	0·92 (0·30–2·29)	0·25 (0·20–0·30)	0·71 (0·65–0·79)	0·69 (0·53–0·96)
	*I^2^*	96% (93–97)	..	..	..	..	..	..
Age†								
	<15 years	0·44 (0·31–0·64)	0·46 (0·30–0·71)	1·29 (0·06–6·38)	..	0·30 (0·14–0·60)	2·23 (0·00–9·59)	1·13 (0·14–4·90)
	15–44 years	0·54 (0·50–0·58)	0·47 (0·42–0·54)	0·60 (0·41–0·85)	..	0·23 (0·18–0·29)	0·72 (0·63, 0·81)	0·70 (0·53, 1·05)
	>44 years	0·59 (0·53–0·65)	0·47 (0·40–0·59)	0·44 (0·24–0·75)	..	0·34 (0·24–0·48)	0·71 (0·62–0·81)	0·72 (0·41–1·20)
	p value	..	1·0	0·55	..	0·15	0·91	0·87
Sex								
	Female	0·54 (0·50–0·60)	0·46 (0·39–0·53)	0·73 (0·50–1·09)	1·01 (0·21–3·48)	0·25 (0·19–0·33)	0·75 (0·64–0·86)	0·76 (0·49–1·19)
	Male	0·55 (0·51–0·60)	0·47 (0·40–0·55)	0·38 (0·24–0·58)	0·67 (0·00–6·76)	0·24 (0·18–0·32)	0·70 (0·63–0·79)	0·63 (0·44–1·00)
	p value	..	0·88	0·037	0·92	0·81	0·49	0·55
Enrolment year†‡								
	2018	0·69 (0·61–0·78)	0·56 (0·47–0·67)	0·56 (0·27–1·11)	..	0·31 (0·19–0·47)	1·05 (0·87–1·30)	0·18 (0·00–0·96)
	2019	0·56 (0·50–0·63)	0·44 (0·37–0·55)	0·59 (0·33–0·97)	..	0·13 (0·09–0·19)	0·85 (0·72–0·99)	0·91 (0·32–1·82)
	2020	0·52 (0·46–0·59)	0·47 (0·39–0·63)	0·56 (0·34–1·02)	..	0·19 (0·11–0·28)	0·60 (0·51–0·71)	0·84 (0·44–1·56)
	2021	0·43 (0·37–0·49)	0·36 (0·27–0·48)	0·51 (0·23–1·01)	..	0·56 (0·36–0·80)	0·44 (0·37–0·53)	0·34 (0·19–0·57)
	2022	0·41 (0·29–0·59)	..	..	..	0·38 (0·23–0·58)	..	0·46 (0·29–0·90)
	p value	..	0·042	0·99	..	<0·0001	<0·0001	0·21
Clinical stage†§								
	Stage 1	0·45 (0·38–0·53)	0·59 (0·47–0·73)	0·96 (0·44–2·30)	..	0·24 (0·19–0·31)	..	0·98 (0·57–1·74)
	Stage 2	0·40 (0·33–0·49)	0·38 (0·37–0·60)	0·50 (0·31–0·86)	..	0·30 (0·17–0·44)	..	0·86 (0·45–1·61)
	Stage 3	0·45 (0·37–0·53)	0·50 (0·31–0·48)	0·51 (0·30–0·80)	..	0·25 (0·15–0·40)	..	0·40 (0·18–0·79)
	Stage 4	0·46 (0·38–0·57)	0·52 (0·40–0·62)	0·29 (0·10–0·63)	..	0·25 (0·14–0·45)	..	0·60 (0·12–2·20)
	p value	..	0·047	0·30	..	0·91	..	0·27
Antiretroviral therapy regimen†								
	Dolutegravir-based	0·55 (0·51–0·60)	0·43 (0·37–0·50)	0·45 (0·30–0·69)	..	0·34 (0·25–0·43)	0·70 (0·62–0·79)	0·80 (0·55–1·40)
	Efavirenz-based	0·62 (0·55–0·69)	0·70 (0·57–0·87)	0·44 (0·26–0·74)	..	0·20 (0·13–0·28)	0·70 (0·60–0·81)	1·10 (0·46–2·16)
	Other	0·52 (0·43–0·63)	0·51 (0·37–0·65)	0·24 (0·00–1·25)	..	0·21 (0·13–0·30)	0·93 (0·66–1·28)	0·46 (0·24–0·83)
	p value	..	0·0012	0·98	..	0·046	0·26	0·18

Data are HR (95% CI), unless otherwise stated. The p value evaluates whether there is evidence that the preventive effectiveness of TPT is different by the measured characteristic. TPT=tuberculosis preventive treatment.

* Did not meet protocol-specified sample size. Results included for completeness.

† Insufficient cases to estimate by subgroup for Nigeria.

‡ 2022 data were not extracted for Haiti, Kenya, Nigeria, or Ukraine.

§ Not reported in Ukraine data.

All countries had increased estimated effect size for reducing tuberculosis incidence and mortality with earlier TPT initiation. For brevity, we present only the results using a 2-week grace period to show this trend (appendix 3 pp 26–33). Adjusted HRs for tuberculosis ranged from 0·25 (95% CI 0·20–0·32) in Haiti to 0·40 (0·23–0·63) in Zimbabwe, with a pooled HR of 0·32 (0·28–0·36), a 7% increase in protection compared with an 8-week grace period. Adjusted HRs for death ranged from 0·20 (0·13–0·28) in Uganda to 0·63 (0·57–0·69) in Ukraine, with a pooled HR of 0·52 (0·49–0·56), a 3% increase in protection compared with an 8-week grace period. Similarly decreased, yet still protective, effectiveness was observed with longer grace periods. No substantial differences were observed by varying the length of the early tuberculosis diagnosis exclusion period.

## Discussion

This study provides the largest real-world evaluation of TPT effectiveness, encompassing over 230 000 people with HIV across six high-tuberculosis and high-HIV-burden settings. We applied an innovative analytical approach for causal inference using observational data to emulate a clinical trial for each country. Across a range of epidemiological and programmatic settings, starting TPT within 8 weeks of entry into HIV care was consistently associated with lower tuberculosis incidence and mortality, with a 61% reduction in tuberculosis incidence and 45% reduction in mortality on average. These findings remained robust for key subgroups, including children and adolescents, and people with HIV receiving dolutegravir-based regimens. These findings reinforce TPT as a crucial intervention for all people with HIV, even in the context of early ART initiation and widespread dolutegravir use.

Despite substantial progress in the global HIV response over the last two decades, tuberculosis remains the leading cause of morbidity and mortality in people with HIV.^1^ Yet, tuberculosis has been preventable with a low-cost, safe drug for over 70 years, which has been included in WHO recommendations for people with HIV since 2011.^29^ The initial focus for HIV programmes in the early 2000s was on increasing knowledge of HIV status and initiating ART, which resulted in substantial reductions in mortality. Although uptake of other prophylactics, such as co-trimoxazole for bacterial infections, was widely implemented, isoniazid for tuberculosis prevention lagged due to myriad concerns, such as the inability to definitively rule out active tuberculosis, emergence of drug resistance, and pill burden when combined with ART.^12^ Even as trials showed that isoniazid reduced mortality among people with HIV on ART, including those with higher CD4 cell counts, use was not widespread until PEPFAR committed to providing TPT to all people with HIV in care in 2018. Following this global mobilisation, over 13 million people with HIV received at least one course of TPT through PEPFAR supported programmes by 2023, making this the only global tuberculosis target from the 2018 UNHLM that was achieved.^1^, ^13^ Key to this success was overcoming barriers to programmatic implementation, such as supply chain management, integration of TPT within HIV service delivery, and stakeholder engagement. Given global funding challenges, ensuring continued vigilance for TPT delivery to people with HIV will be crucial to ensure gains are sustained.

Evidence from clinical trials can be used to guide practice, but real-world impact can vary due to heterogeneous patient populations, screening procedures, inadequate follow-up, other comorbidities that are often exclusionary or intensively managed in trial settings, and implementation challenges in programmatic settings. Our study examined the programmatic effectiveness of TPT using observational data at national and regional levels in countries with varying epidemiological profiles. By applying a target trial design to harmonise inherent variability in observational data from over 230 000 people with HIV, we showed substantially lower tuberculosis incidence and mortality in those who initiated TPT. We estimated an intent-to-treat effect of TPT that incorporates non-adherence and treatment discontinuation as they occur in routine care. In line with this pragmatic approach, we allowed up to 8 weeks for TPT initiation to further accommodate other practical factors that can affect TPT initiation including diagnostic evaluation, medication availability, and competing clinical priorities such as ART adherence. Although sensitivity analyses suggested increased protection with initiation within 2 weeks, meaningful benefit was still observed when initiation occurred within 8 weeks. Because our analysis explicitly accommodates real-world programmatic constraints, our results are probably conservative and might underestimate the biological effect of TPT in settings with immediate initiation and full adherence among people with HIV.

The protective effect of TPT was robust across subgroups, including children younger than 15 years with HIV, people with early-stage HIV disease, and those receiving dolutegravir-based regimens. For children with HIV, TPT coverage has lagged due to additional challenges with drug supply for weight-based dosing, adherence concerns, and various modifiable factors.^30^ People with early-stage HIV, as determined by WHO staging, are a crucial group for tuberculosis prevention. Despite imperfections with the WHO staging approach, our data support TPT across the spectrum of HIV disease presentation at entry into care, including people with HIV classified with early-stage disease. Finally, dolutegravir-based regimens initially raised concerns about drug–drug interactions when co-administered with TPT, particularly regimens containing rifapentine. However, a 2025 trial showed the safety and efficacy of initiating treatment concurrently.^31^ Our analytical approach and sensitivity analysis allowed for variation in the grace period for TPT initiation based on programme realities or requirements and found TPT was most beneficial when started early.

Our study has several limitations. First, we relied on data recorded in EMRs, which could have been incomplete. Missingness was minimised through back-capture of data from multiple paper records; however, the validity of our analysis relies on an assumption that missingness can be appropriately accounted for using measured covariates, which might not be true. Although CD4 cell count was missing for a large proportion of people with HIV (other than in Kenya and Ukraine), the proportion who initiated TPT did not differ based on degree of immunosuppression, where data were available. Second, unmeasured factors could have affected who received TPT and when, including drug supply, national policies prioritising newly diagnosed people with HIV over those stable in care, tuberculosis screening and diagnosis, and provider and patient preferences, among others that could have been exacerbated during the COVID-19 pandemic. Importantly, tuberculosis care remained a priority during the COVID-19 pandemic, including ensuring people with HIV received and completed TPT. Our large, national or nationally and regionally representative cohorts spanning a 4-year period sought to capture a representative study population to account for these variations. However, bias in effectiveness estimates might remain due to unmeasured confounding by indication, particularly if healthier people with HIV preferentially received TPT. Precision of estimates for children with HIV was limited due to small sample size. Third, we did not ascertain data on TPT adherence as data on TPT completion dates were not considered sufficiently reliable in most countries. Thus, our results describe the effectiveness only of TPT initiation and could be considered conservative for the effectiveness of TPT completion. Finally, the sample size in Nigeria was not sufficient to evaluate country-level outcomes with adequate precision. However, the direction of the effect was consistent with that observed in other countries and the inclusion of Nigeria in the pooled meta-analysis is unlikely to alter results meaningfully as pooled estimates were inverse-variance weighted, thereby appropriately decreasing the influence of the underpowered effect estimate.

In conclusion, TPT was effective in reducing tuberculosis incidence and mortality in people with HIV across various epidemiological contexts and risk groups. These data reaffirm findings from previous clinical trials but provide the largest programmatic assessment of real-world impact. As short TPT regimens become increasingly available, uptake and completion rates are likely to improve. Additional research to evaluate short TPT regimens and the duration of protective effect beyond 2 years is needed. In an era of universal test-and-treat and transitioning global programmes, tuberculosis prevention remains an essential component of HIV care.

For **analysis code** see https://github.com/benkeser/protectr

### Contributors

### Data sharing

De-identified data can be made available upon reasonable request, with required approvals and data use agreements from the country's Ministry of Health and participating implementing partners, who are the owners of the data. Proposals should be directed to sarita.shah@emory.edu. Due to ethical restrictions, data requestors will need to sign a data access agreement to gain access.

## Declaration of interests

We have no competing interests to declare.

## Supplementary Materials

French translation of the abstract

Ukrainian translation of the abstract
