# Supplementary material for: Programmatic effectiveness of tuberculosis preventive treatment for people with HIV in six high-tuberculosis-burden countries (PROTECT): a prospective cohort study and meta-analysis
Source: Lancet HIV. 2026 Aug 17;13(10):e723–34. doi: 10.1016/S2352-3018(26)00140-2 (PMC13626977; doi:10.1016/S2352-3018(26)00140-2)
Supplement: Supplementary file 1 [file mmc1.pdf]

THE LANCET
HIV

Supplementary appendix 1

This translation in French was submitted by the authors and we reproduce it as supplied. It has not been peer reviewed. *The Lancet*'s editorial processes have only been applied to the original in English, which should serve as reference for this manuscript.

Cette traduction en français a été proposée par les auteurs et nous l'avons reproduite telle quelle. Elle n'a pas été examinée par des pairs. Les processus éditoriaux du *Lancet* n'ont été appliqués qu'à l'original en anglais et c'est cette version qui doit servir de référence pour ce manuscrit.

Supplement to: Shah NS, Mishara F, Zissette S, et al. Programmatic effectiveness of tuberculosis preventive treatment for people with HIV in six high-tuberculosis-burden countries (PROTECT): a prospective cohort study and meta-analysis. *Lancet HIV* 2026; published online Aug 17. https://doi.org/10.1016/S2352-3018(26)00140-2.

Contexte: Les essais cliniques randomisés (ECR) ont démontré que le traitement préventif de la tuberculose (TPT) réduit l'incidence de la maladie et la mortalité chez les personnes vivant avec le VIH (PVVIH). Cependant, l'impact dans les conditions programmatiques n'a pas été évalué depuis que l'éligibilité au traitement antirétroviral (TAR) a été étendue à toutes les PVVIH, quel que soit leur taux de lymphocytes CD4. L'objectif de cette étude était de déterminer l'efficacité du TPT sur la réduction de l'incidence de la tuberculose et de la mortalité toutes causes confondues chez les PVVIH débutant un TAR.

Méthodes: Cette étude de cohorte prospective a émulé six ECR en utilisant des données individuelles issues de dossiers médicaux électroniques en Haïti, Ouganda, Ukraine, Zimbabwe, Kenya et au Nigéria. L'évaluation a porté sur l'efficacité du TPT chez les PVVIH débutant un TAR entre le 1er janvier 2018 et le 31 décembre 2021 (Haïti, Kenya, Nigéria et Ukraine) ou le 31 décembre 2022 (Ouganda et Zimbabwe). Dans chaque pays, nous avons constitué des cohortes de PVVIH débutant le TAR, quel que soit leur âge. Nous avons exclu les personnes ayant reçu un diagnostic de tuberculose 6 mois avant et 30 jours après l'initiation du TAR. Les données extraites anonymisées comprenaient des variables pour chaque visite entre la date d'enrôlement en soins VIH et le 31 décembre 2023 (Haïti, Kenya, Nigéria et Ukraine) ou le 31 décembre 2024 (Ouganda et Zimbabwe). La méthode d'émulation d'un essai clinique cible a été utilisée pour définir un essai randomisé hypothétique, comparant un groupe recevant le TPT pendant une période de grâce de 8 semaines à un groupe n'ayant pas recu de TPT. L'efficacité du TPT sur l'incidence de la tuberculose et la mortalité toutes causes confondues a été estimée à l'aide de rapports de risques instantanés ajustés (RRa). Cette estimation a utilisé l'approche de pondération par l'inverse de la probabilité de traitement, avec un ajustement pour les variables confondantes (âge, sexe, taux de CD4, charge virale, stade clinique, initiation et observance au TAR). Les résultats des six pays ont été regroupés dans une méta-analyse en utilisant la pondération par l'inverse de la variance, l'hétérogénéité des effets étant évaluée à l'aide de la statistique I^2.

Résultats: Nous avons inclus 235 424 PVVIH : 69 936 personnes d'Haïti, 65 936 d'Ouganda, 57 851 de l'Ukraine, 24 099 du Zimbabwe, 9 549 du Kenya et 8 053 du Nigeria. Sur les 235 424 personnes, 147 667 (62,7 %) étaient âgées de 25 à 44 ans, 133 582 (56,7 %) étaient de sexe féminin, 101 842 (43,3 %) étaient de sexe masculin, 104 277 (44,3 %) présentaient un stade clinique 1, et 138 914 (59,0 %) suivaient des schémas thérapeutiques à base de dolutégravir. Le RRa pour la tuberculose variait de 0,35 (IC à 95% : 0,28–0,43) en Haïti à 0,60 (IC à 95% : 0,36–0,91) au Kenya. Le RR global pour la tuberculose était de 0,39 (IC à 95% : 0,34–0,43) avec une variation minime selon les pays (I^2 = 0%, IC à 95% : 0%–75%). Le RRa pour la mortalité allait de 0,25 (IC à 95% 0,20–0,30) en Ouganda à 0,69 (IC à 95% : 0,53–0,96) au Zimbabwe. Le RR global pour la mortalité était de 0,55 (IC à 95% : 0,51–0,58) avec une variation significative d'un pays à l'autre (I^2 = 96%, IC à 95% : 93%–97%).

Conclusion: Le TPT a réduit l'incidence de la tuberculose et la mortalité chez les PVVIH dans des groupes variés et à travers divers contextes épidémiologiques. À l'ère du traitement universel (*test-and-treat*) et des nouveaux schémas de TAR, ces résultats renforcent le rôle essentiel du TPT dans la prise en charge du VIH.
