# Supplementary material for: Programmatic effectiveness of tuberculosis preventive treatment for people with HIV in six high-tuberculosis-burden countries (PROTECT): a prospective cohort study and meta-analysis
Source: Lancet HIV. 2026 Aug 17;13(10):e723–34. doi: 10.1016/S2352-3018(26)00140-2 (PMC13626977; doi:10.1016/S2352-3018(26)00140-2)
Supplement: Supplementary file 2 [file mmc2.pdf]

THE LANCET
HIV

## Supplementary appendix 2

This translation in Ukrainian was submitted by the authors and we reproduce it as supplied. It has not been peer reviewed. *The Lancet*'s editorial processes have only been applied to the original in English, which should serve as reference for this manuscript.

Цей переклад українською мовою був наданий авторами, і ми відтворюємо його в тому вигляді, в якому він був наданий. Переклад не проходив рецензування. Редакційні процеси журналу Lancet були застосовані лише до оригіналу англійською мовою, на який слід посилатися як на джерело при цитуванні.

Supplement to: Shah NS, Mishara F, Zissette S, et al. Programmatic effectiveness of tuberculosis preventive treatment for people with HIV in six high-tuberculosis burden countries (PROTECT): a prospective cohort study and meta-analysis. *Lancet HIV* 2026; published online Aug 17. https://doi.org/10.1016/S2352-3018(26)00140-2.

**РЕЗЮМЕ**

**Передумови:** У рандомізованих клінічних дослідженнях (РКД) профілактичне лікування туберкульозу (ПЛТ) знижувало захворюваність на туберкульоз (ТБ) та смертність серед людей, які живуть з ВІЛ (ЛЖВ). Однак його вплив у реальних умовах ще не оцінювався після розширення антиретровірусної терапії (АРТ) для всіх ЛЖВ незалежно від рівня CD4-клітин. Це дослідження мало на меті визначити програмну ефективність ПЛТ щодо зниження захворюваності на ТБ та смертності від усіх причин серед ЛЖВ, які розпочинають АРТ.

**Методи:** У цьому проспективному когортному дослідженні ми емулювали шість РКД, використовуючи індивідуальні дані з електронних медичних записів у Гаїті, Кенії, Нігерії, Уганді, Україні та Зімбабве, щоб оцінити ефективність ПЛТ серед ЛЖВ, які розпочали АРТ у період з 1 січня 2018 року по 31 грудня 2021 року (Гаїті, Кенія, Нігерія та Україна) або по 31 грудня 2022 року (Уганда та Зімбабве). У кожній країні було сформовано когорти ЛЖВ, які вперше розпочали АРТ, незалежно від віку. Ми виключили осіб, у яких ТБ було діагностовано в період від 6 місяців до початку АРТ до 30 днів після її початку. Для кожного пацієнта використовувалися деперсоналізовані дані щодо вибраних змінних на рівні візитів за період від включення до ВІЛ-допомоги до 31 грудня 2023 року (Гаїті, Кенія, Нігерія та Україна) або до 31 грудня 2024 року (Уганда та Зімбабве). Для визначення гіпотетичного рандомізованого дослідження застосовували підхід емулювання цільового дослідження (target trial emulation), порівнюючи початок ПЛТ протягом 8-тижневого пільгового періоду з відсутністю початку ПЛТ. Ефективність ПЛТ оцінювали за допомогою скоригованих коефіцієнтів ризику (aHR) розвитку ТБ та смертності від усіх причин, розрахованих із застосуванням підходу зворотного зважування за схильністю (inverse propensity weighting), із корекцією на змішані фактори, включаючи вік і стать (незмінні в часі), а також рівень CD4, вірусне навантаження, клінічну стадію, початок АРТ та прихильність до АРТ (змінні в часі). Дані шести країн були об'єднані в метааналізі із застосуванням зворотного зважування за дисперсією; гетерогенність оцінювали за показником $I^2$.

**Результати:** До дослідження було включено 235 424 ЛЖВ: Гаїті (n=69 936), Уганда (n=65 936), Україна (n=57 851), Зімбабве (n=24 099), Кенія (n=9 549) та Нігерія (n=8 053). Більшість були віком 25–44 роки (62,7%), жінки (56,7%), мали клінічну стадію 1 (44,3%) та отримували схеми лікування на основі долутегравіру (59,0%). Скоригований коефіцієнт ризику розвитку ТБ коливався від 0,35 (95% ДІ 0,28–0,43) у Гаїті до 0,60 (95% ДІ 0,36–0,91) у Кенії. Сукупний aHR для ТБ становив 0,39 (95% ДІ 0,34–0,43) з мінімальними відмінностями між країнами ($I^2$ = 0%, 95% ДІ 0–75%). Скоригований коефіцієнт ризику смертності коливався від 0,25 (95% ДІ 0,20–0,30) в Уганді до 0,69 (95% ДІ 0,53–0,96) у Зімбабве. Сукупний коефіцієнт ризику смертності становив 0,55 (95% ДІ 0,51–0,58) зі значною варіабельністю між країнами ($I^2$ = 96%, 95% ДІ 93–97%).

**Висновок:** ПЛТ було ефективним у зниженні захворюваності на ТБ та смертності серед ЛЖВ у різних епідеміологічних умовах і групах ризику. В епоху універсального підходу «test-and-treat» та нових схем АРТ ці дані підтверджують, що ПЛТ залишається важливим компонентом ВІЛ-допомоги.
