# Supplementary material for: Programmatic effectiveness of tuberculosis preventive treatment for people with HIV in six high-tuberculosis-burden countries (PROTECT): a prospective cohort study and meta-analysis
Source: Lancet HIV. 2026 Aug 17;13(10):e723–34. doi: 10.1016/S2352-3018(26)00140-2 (PMC13626977; doi:10.1016/S2352-3018(26)00140-2)
Supplement: Supplementary file 3 [file mmc3.pdf]

THE LANCET
HIV

Supplementary appendix 3

This appendix formed part of the original submission and has been peer reviewed. We post it as supplied by the authors.

Supplement to: Shah NS, Mishara F, Zissette S, et al. Programmatic effectiveness of tuberculosis preventive treatment for people with HIV in six high-tuberculosis-burden countries (PROTECT): a prospective cohort study and meta-analysis. *Lancet HIV* 2026; published online Aug 17. https://doi.org/10.1016/S2352-3018(26)00140-2.

Supplementary Appendix

Programmatic effectiveness of tuberculosis preventive treatment for people with HIV in six high tuberculosis burden countries: An observational cohort study using target trial emulation

Table of Contents

Authors 3
Supplemental Table S1. HIV and TB epidemic and program characteristics of PROTECT study countries 4
Supplemental Methods: Data quality assessment. 6
Supplemental Table S2. Adjustment covariates used in propensity score modeling for each country 8
Supplemental Table S3. Person time contributions for different types of patients in the clone-censor-weight analysis. 9
Supplemental Figure S1. Patient inclusion flow chart for Haiti study cohort 10
Supplemental Figure S2. Patient inclusion flow chart for Kenya study cohort 10
Supplemental Figure S3. Patient inclusion flow chart for Nigeria study cohort 11
Supplemental Figure S4. Patient inclusion flow chart for Uganda study cohort 11
Supplemental Figure S5. Patient inclusion flow chart for Ukraine study cohort 12
Supplemental Figure S6. Patient inclusion flow chart for Zimbabwe study cohort 12
Supplemental Table S4. Demographic characteristics of Haiti study cohort by observed TPT status. 13
Supplemental Table S5. Demographic characteristics of Kenya study cohort by observed TPT status. 15
Supplemental Table S6. Demographic characteristics of Nigeria study cohort by observed TPT status 17
Supplemental Table S7. Demographic characteristics of Uganda study cohort by observed TPT status 19
Supplemental Table S8. Demographic characteristics of Ukraine study cohort by observed TPT status 21
Supplemental Table S9. Demographic characteristics of Zimbabwe study cohort by observed TPT status 23
Supplemental Table S10. Person time contributions for estimation of TPT effectiveness across countries. 25
Supplemental Figure S7. Two-year cumulative incidence (95% CI) of TB under each treatment scenario by country comparing TPT initiation within 2 weeks of enrollment into care to never initiating TPT 26

Supplemental Figure 8. Two-year cumulative incidence (95% CI) of all-cause mortality under each treatment scenario by country comparing TPT initiation within 2 weeks of enrollment into care to never initiating TPT 27

Supplemental Table S12. Adjusted hazard ratios for tuberculosis diagnosis comparing TPT initiation within 2 weeks of enrollment into care to never initiating TPT, pooled and by country....28

Supplemental Table S13. Adjusted hazard ratios for all-cause mortality comparing TPT initiation within 2 weeks of enrollment into care to never initiating TPT, pooled and by country....29

Supplemental Figure S9. Adjusted hazard ratios of tuberculosis diagnosis comparing TPT initiation within 2 weeks of enrollment into care to never initiating TPT, pooled and by country....30

Supplemental Figure S10. Adjusted hazard ratios of all-cause mortality comparing TPT initiation within 2 weeks of enrollment into care to never initiating TPT, pooled and by country....30

Supplemental Figure S11. Pooled adjusted hazard ratios of tuberculosis diagnosis comparing TPT initiation within 2 weeks of enrollment into care to never initiating TPT, overall and within population subgroups....31

Supplemental Figure S12. Pooled adjusted hazard ratios of all-cause mortality comparing TPT initiation within 2 weeks of enrollment into care to never initiating TPT, overall and within population subgroups....32

REFERENCES....32

Authors:

N. Sarita Shah, MD; Fadimatu Mishara, MD; Seth Zissette, MS; Nephtalie Mesidor, MD; Solomon Ssevvume, MS; Serhii Riabokon, MD; Blessing Mushangwe, MBChB ; John Motoku, MBBS; Stephen Balogun, MD; Nassir Kashmiri, BS; Maryam Bello, MPH; Augustin Gerry Marcel, MD; Deus Lukoye, PhD; Ezra Barzilay, MD; Talent Maphosa, MDS; Herman Weyenga, MBChB; Ademola Oladipo, MD; Odile Ferroussier-Davis, PhD; Juliana da Silva, MD; Julia Ershova, PhD; Aderonke S. Ajiboye, MPH; Brigette L. Gleason, MD; Jean Solon Valles, MS; Kurt Jean-Charles, MS; Thomas Achia, PhD; David Benkeser, PhD; Macarthur Charles, MD; on behalf of the PROTECT Study team*

*PROTECT Study team:

- CDC Foundation: Reema Bhakta
- Haiti: Kesner François, Dianne Jean-Francois, Celestin Nicasky, Milo Richard, Jean Gregory Solide
- Uganda: Ibrahim Lutalo, Simon Muchuro, Proscovia Namuwenge, Simon Walusimbi
- Ukraine: Larysa Hetman, Petro Martynov, Sherri Pals, Nataliya Podolchak, Olutomi Sodeke
- Zimbabwe: Simbareshe Chaputsira, Rumbidizai Dhliwayo, Gloria Gonese, Danai Madzivire, Chiedza Marisa, Wadzanai Munakamwe,
- Kenya: Wilson Waithaka
- Nigeria: Samuel Abujah, Minal Ahson, Chukwuma Anyaike, Kehinde Balogun, Adebobola Bashorun, Isa Daniel, Victor Temitope Falokun, Ibrahim Bola Gobir, Francis Muoghalu, Pringar Niyang, Flora Nwagagbo, Nnamdi Umeh

Supplemental Table S1. HIV and TB epidemic and program characteristics of PROTECT study countries

	**Haiti**	**Kenya**	**Nigeria**	**Uganda**	**Ukraine**	**Zimbabwe**
Estimated PWH, nationwide (2023)[1]	140,000	1.4 million	2.0 million	1.5 million	158,800	1.3 million
PWH who know their status (%)[1]	87%	96%	85%	93%	100%	95%
PWH receiving ART, nationwide (%)[1]	117,761 (84%)	1.34 million (94%)	1.74 million (85%)	1.24 million (83%)	126,000 (80%)	1.23 million (95%)
Treat all (universal ART) widescale implementation[1]	2017	2018	2017	2017	2016	2017
PWH with suppressed viral load (HIV-1 RNA <1,000 copies per mL)[1]	71%	91%	82%	77%	87%	91%
Estimated TB incidence, 2024 (per 100,000 population)[2]	175	207	219	197	86	203
TB incidence in PWH (2024 year for data source)[2]	26 per 100,000	44 per 100,000	11 per 100,000	65 per 100,000	17 per 100,000	121 per 100,000
TB screening policy for PWH	4 symptom screen at every visit	4 symptom screen at every visit	4 symptom screen at every visit	4 symptom screen at every visit	4 symptom screen at every visit	4 symptom screen at every visit
TB diagnostic evaluation	mWRD<br>Microscopy<br>CXR	mWRD<br>Microscopy (with specimen referral for mWRD)<br>CXR	mWRD (microscopy if not accessible), imaging, histological test	mWRD<br>TB-LAM (advanced HIV)<br>CXR (+/- CAD)	mWRD<br>Microscopy<br>CXR	mWRD<br>TB-LAM<br>CXR
TB treatment regimen	4HRZE/2HR	4HRZE/2HR	4HRZE/2HR	4HRZE/2HR	4HRZE/2HR	4HRZE/2HR
TPT widescale implementation	2017	2015	2018	2018	2018	2018
PWH who received at least one course of TPT[3]	121,930	1,185,914	2,344,089	1,483,255	46,076	1,098,391
TPT regimen (2018-2021)	6H	6H	6H	6H (delayed 3 months for PLHIV initiated on DTG-based ART)	6H	6H or 3HP/3HR (delayed 3

						months until mid-2018)
EMR implemented (year)	SALVH (2008) TB Tracker (2018)	Ecare (EDARP, 2004)	NMRS, 2004	UgandaEMR, 2011 ClinicMaster	2004 ICS	2016 (Impilo EHR)
EMR coverage, number of ART facilities (%)	165 (90%)	1,516 (50%)	2,004 (100%)	1,827 (72%)	551 (100%)	1,050 (56%)

PWH: people living with HIV; ART: antiretroviral therapy; TB: tuberculosis; TPT: tuberculosis preventive treatment; EMR: electronic medical record; mWRD: molecular WHO-recommended rapid diagnostic test; CXR: chest radiography; CAD: computer-aided detection; TB-LAM: tuberculosis lipoarabinomannan; 4HRZE/2HR: 4 months isoniazid, rifampin, pyrazinamide, ethambutol and 2 months isoniazid plus rifampin; 6H: 6 months isoniazid

Supplemental Methods: Data quality assessment

Data quality assessments were conducted by the PROTECT study team in Uganda, Nigeria, and Zimbabwe at the facility level with systematic cross-checking of electronic medical record (EMR) data against source registers. Routine data quality assessment is conducted by the HIV and TB program in Haiti, Kenya, and Ukraine so was not done specifically for the PROTECT study. Record-level validation covered key variables, including TPT start and completion, TB diagnosis, death, ART initiation, viral load testing, and patient outcomes (e.g., transfer and loss to follow up). In Uganda, a total of 69,281 patient records were reviewed. Approximately 10% discrepancy rate was observed, largely due to delays in updating EMR data from paper-based registers. Discrepancies were resolved through site-level verification and reconciliation using a structured tracking tool, with source registers treated as the reference standard. In Nigeria, a total of 18,214 patient records were reviewed. A higher discrepancy rate of ~35% was identified, primarily reflecting gaps between EMR and source documentation due to incomplete or delayed data entry. Targeted data quality audits and primary data re-entry at the patient level were conducted to correct discrepancies and improve completeness. In Zimbabwe, a total of 36,028 patient records were reviewed. Approximately 20% discrepancy rate was identified. Discrepancies were addressed through facility-level verification and reconciliation processes using a structured tracking tool, with paper-based registers used as the primary reference source for updating EMR data. Variables of interest for data quality assessments included:

**Baseline visit variables**	**Follow-up visit variables**
Patient ID	Date of follow-up visit
Sex	Prior ART
Age	Current ART regimen
Health facility name	Follow up weight
Date of HIV diagnosis	Medications (including ART)
Date of enrollment in HIV care	TB symptoms at follow-up visit
WHO Clinical stage at enrollment	TPT start date
Weight at enrollment	TPT completion date
Symptoms at enrollment	Date of TB diagnosis
CD4 count at HIV diagnosis	TB diagnosis type
Date of first CD4 count	TB treatment start date
TB status at enrollment	TB treatment outcome
ART start date	Date of TB treatment outcome
ART regimen	Follow-up CD4 count
	Date of follow-up CD4 count

	Follow-up viral load
	Date of follow-up viral load
	ART Follow up status
	Reasons for stopping ART
	Date of next follow-up visit
	Vital status

Supplemental Table S2. Adjustment covariates used in propensity score modeling for each country

Country	Time-Invariant Covariates	Time-Varying Covariates
Haiti	Enrollment year, age, sex, geography (facility district, patient district), clinical stage at enrollment, ART initiation date, weight at enrollment (ages 15 and older only)	Week, visit-level ART adherence, visit-level TB symptoms, visit-level viral load count, visit-level clinical stage
Kenya	Enrollment year, age, sex, geography (subcounty), education level, religion, clinical stage at enrollment, ART initiation date, known TB prior to entry into HIV care, number of healthcare encounters within 8 weeks of entry into HIV care (enrollment years 2018 and 2019 only)	Week, visit-level ART adherence, visit-level CD4 count, visit-level TB symptoms, visit-level viral load count
Nigeria	Enrollment year, age, sex, geography (state), clinical stage at enrollment, ART initiation date, history of high alcohol use	Week, visit-level ART adherence, visit-level CD4 count, visit-level TB symptoms, visit-level viral load count, visit-level clinical stage
Uganda	Enrollment year, age, sex, geography (region), clinical stage at enrollment, ART initiation date	Week, visit-level ART adherence, visit-level CD4 count, visit-level viral load count, visit-level clinical stage
Ukraine	Enrollment year, age, sex, ART initiation date, history of high alcohol use, history of imprisonment	Week, visit-level ART adherence, visit-level CD4 count, visit-level viral load count
Zimbabwe	Enrollment year, age, sex, geography (district), clinical stage at enrollment, ART initiation date	Week, visit-level CD4 count, visit-level viral load count, visit-level clinical stage

ART: antiretroviral therapy; TB: tuberculosis

Supplemental Table S3. Person time contributions for different types of patients in the clone-censor-weight analysis.

TB outcome

Type of patient	Contribution to estimation of TB incidence under TPT intervention	Contribution to estimation of TB incidence under no TPT intervention
TPT initiated in grace period	All weeks of follow up from time of enrollment into HIV care until death, TB diagnosis, or right-censoring event, whichever occurs first.	All weeks of follow up from time of enrollment into HIV care until week of TB diagnosis, week that TPT is initiated, or week of right censoring for TB outcome, whichever occurs first.
TPT initiated outside grace period	All weeks of follow up from time of enrollment into HIV care until death, TB diagnosis, week of right censoring for TB outcome, or the last week of the grace period, whichever occurs first.	All weeks of follow up from time of enrollment into HIV care until death, TB diagnosis, or TPT is initiated, whichever occurs first.
TPT never initiated	All weeks of follow up from time of enrollment into HIV care until death, TB diagnosis, right-censoring event, or the last week of the grace period, whichever occurs first.	All weeks of follow up from time of enrollment into HIV care until death, TB diagnosis, or right-censoring event, whichever occurs first.

All-cause mortality outcome

Type of patient	Contribution to estimation of all-cause mortality incidence under TPT intervention	Contribution to estimation of all-cause mortality incidence under no TPT intervention
TPT initiated in grace period	All weeks of follow up from time of enrollment into HIV care until death or right-censoring for all-cause mortality outcome, whichever occurs first.	All weeks of follow up from time of enrollment into HIV care until week that TPT is initiated or week of right-censoring for all-cause mortality outcome, whichever occurs first.
TPT initiated outside grace period	All weeks of follow up from time of enrollment into HIV care until death, right-censoring for all-cause mortality outcome or the last week of the grace period, whichever occurs first.	All weeks of follow up from time of enrollment into HIV care until death, right-censoring for all-cause mortality outcome or TPT is initiated, whichever occurs first.
TPT never initiated	All weeks of follow up from time of enrollment into HIV care until death, right-censoring event, or the last week of the grace period, whichever occurs first.	All weeks of follow up from time of enrollment into HIV care until first occurrence of death or right-censoring event, whichever occurs first.

Supplemental Figure S1. Patient inclusion flow chart for Haiti study cohort

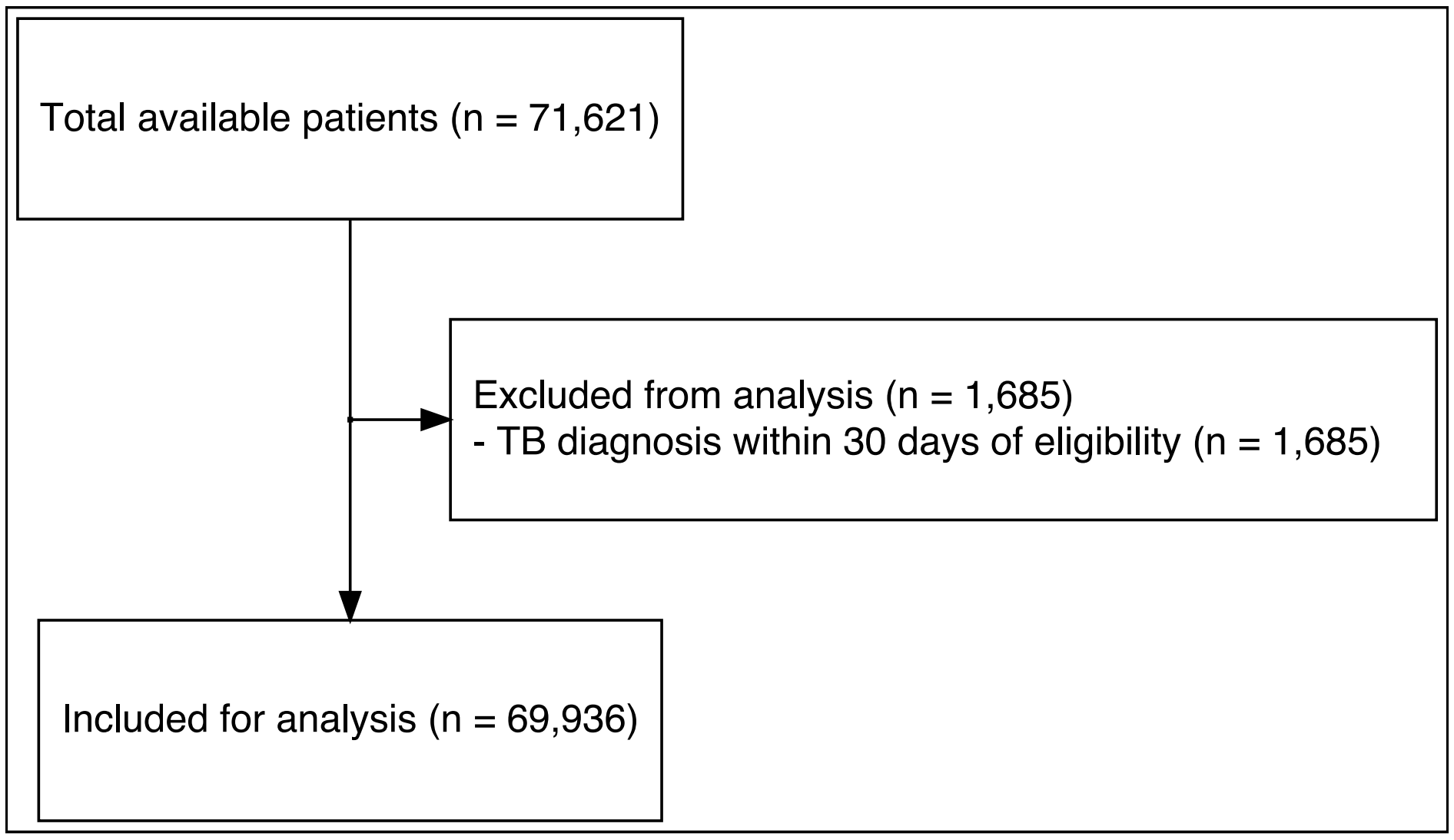

Supplemental Figure S2. Patient inclusion flow chart for Kenya study cohort

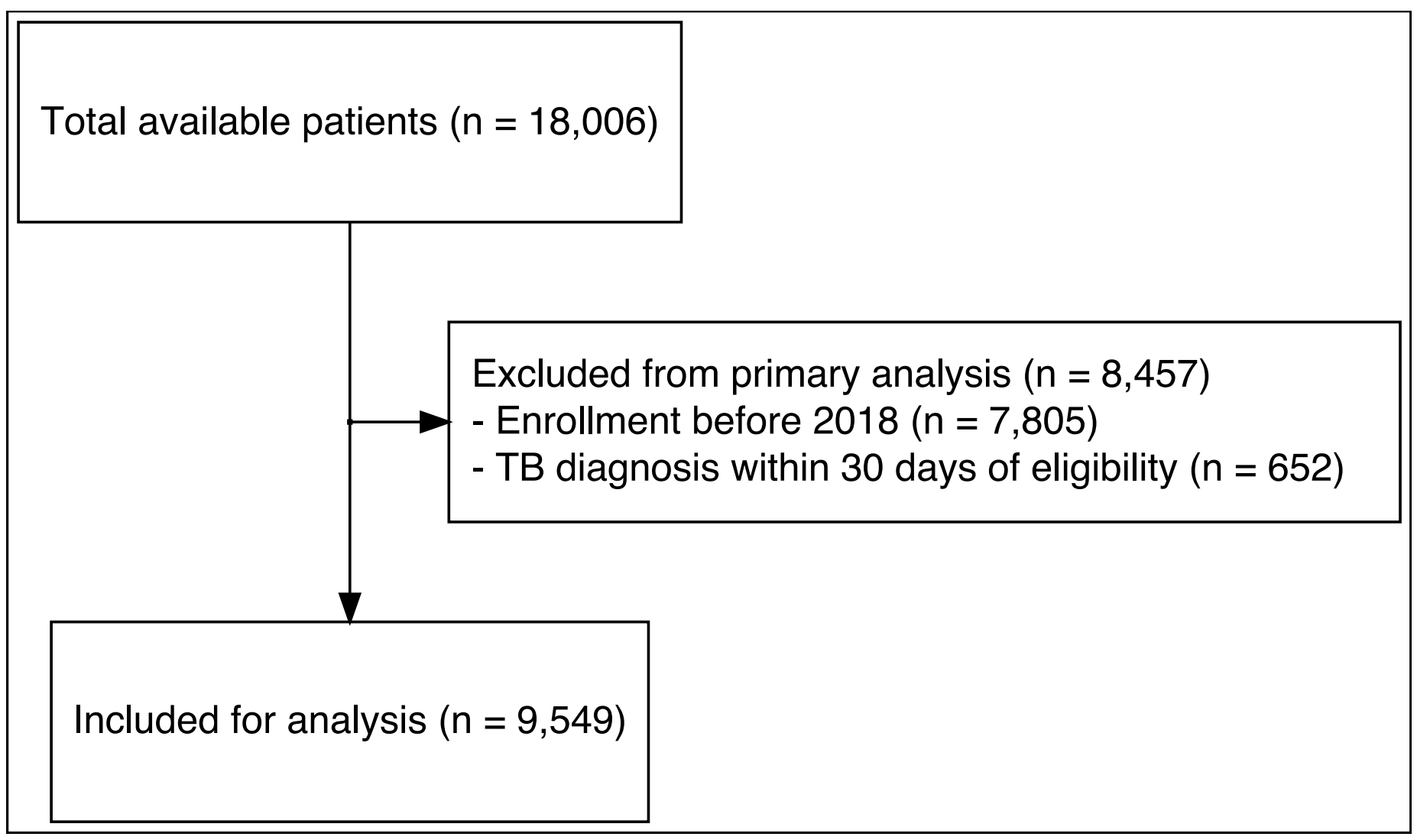

Supplemental Figure S3. Patient inclusion flow chart for Nigeria study cohort

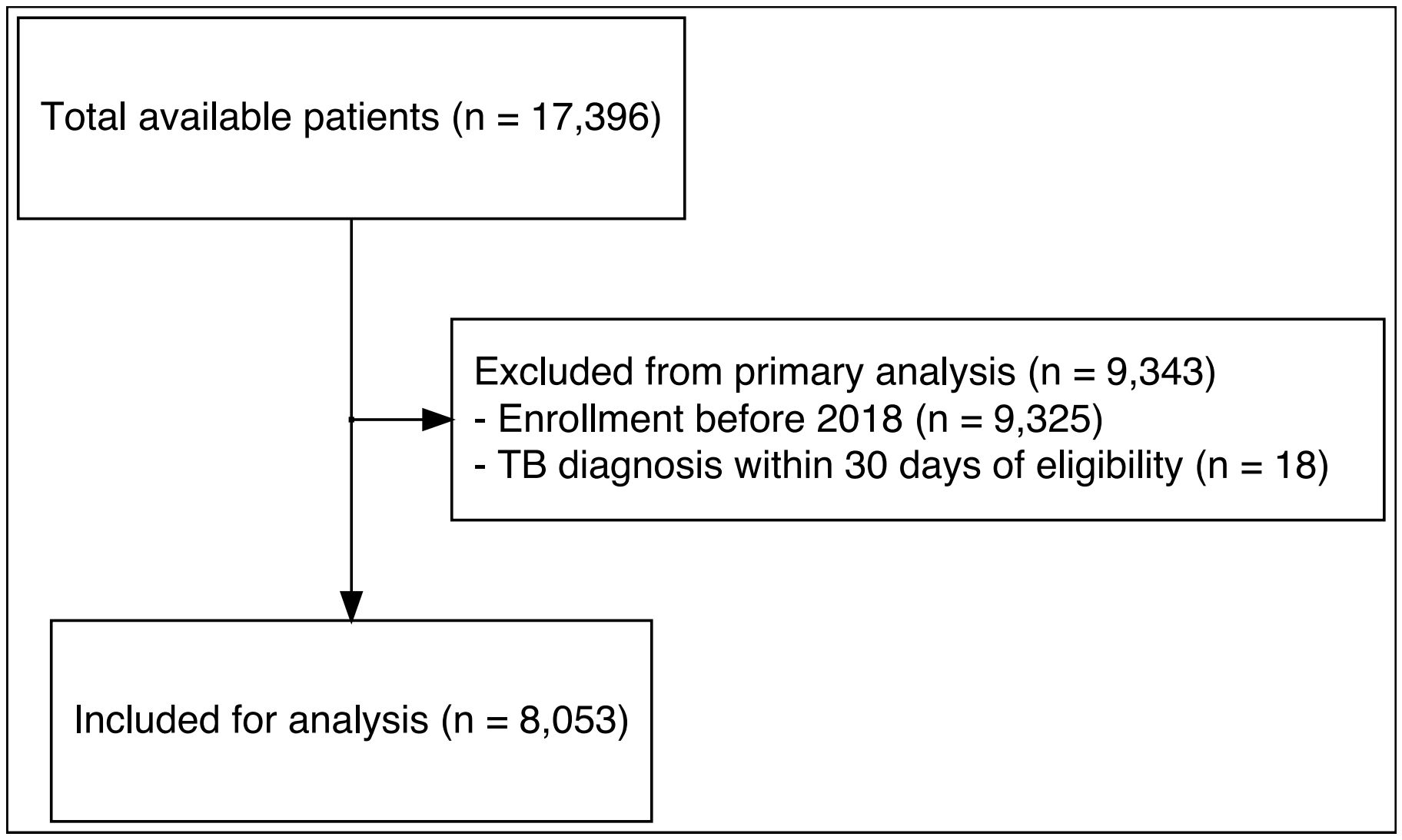

Supplemental Figure S4. Patient inclusion flow chart for Uganda study cohort

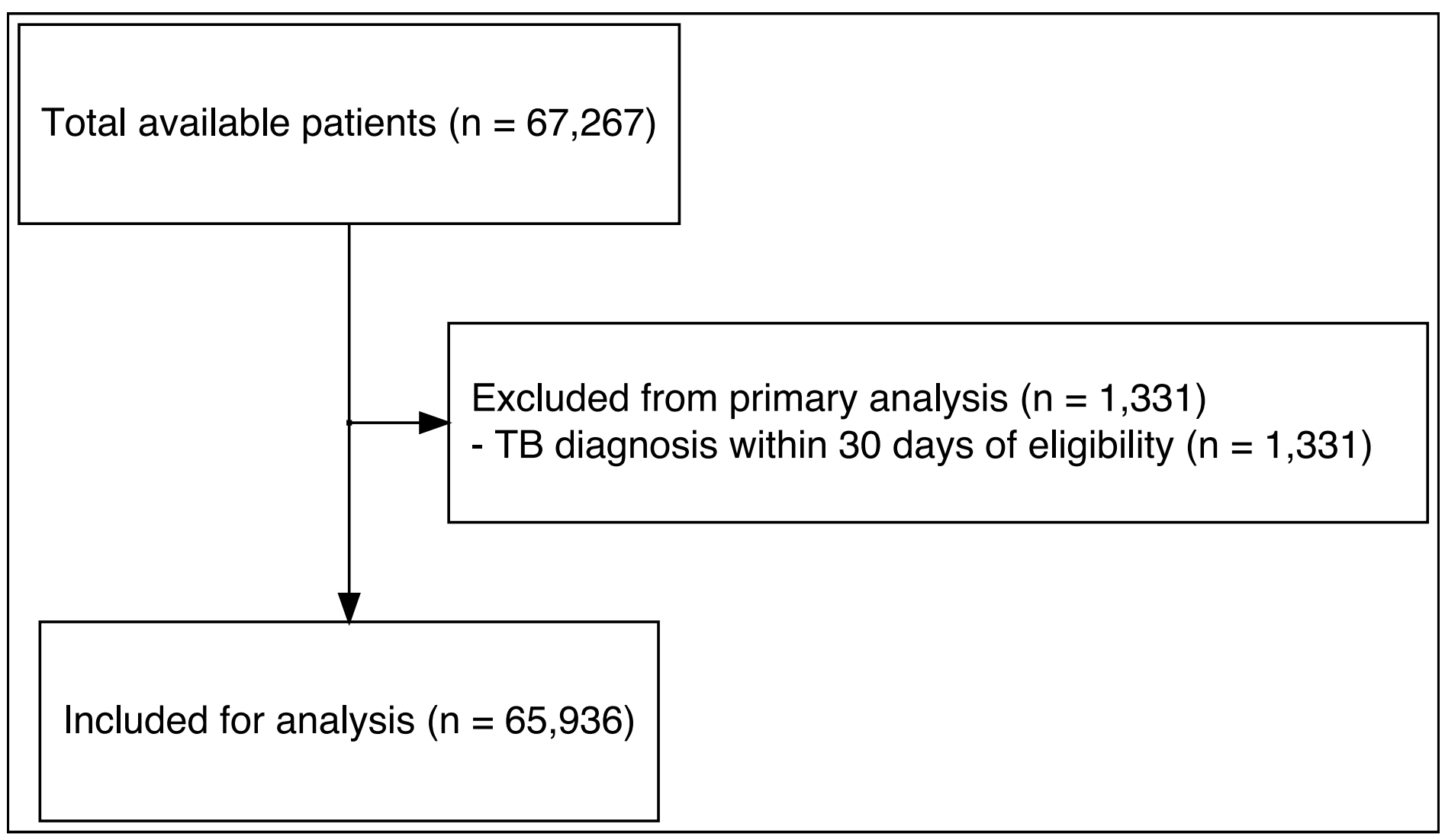

Supplemental Figure S5. Patient inclusion flow chart for Ukraine study cohort

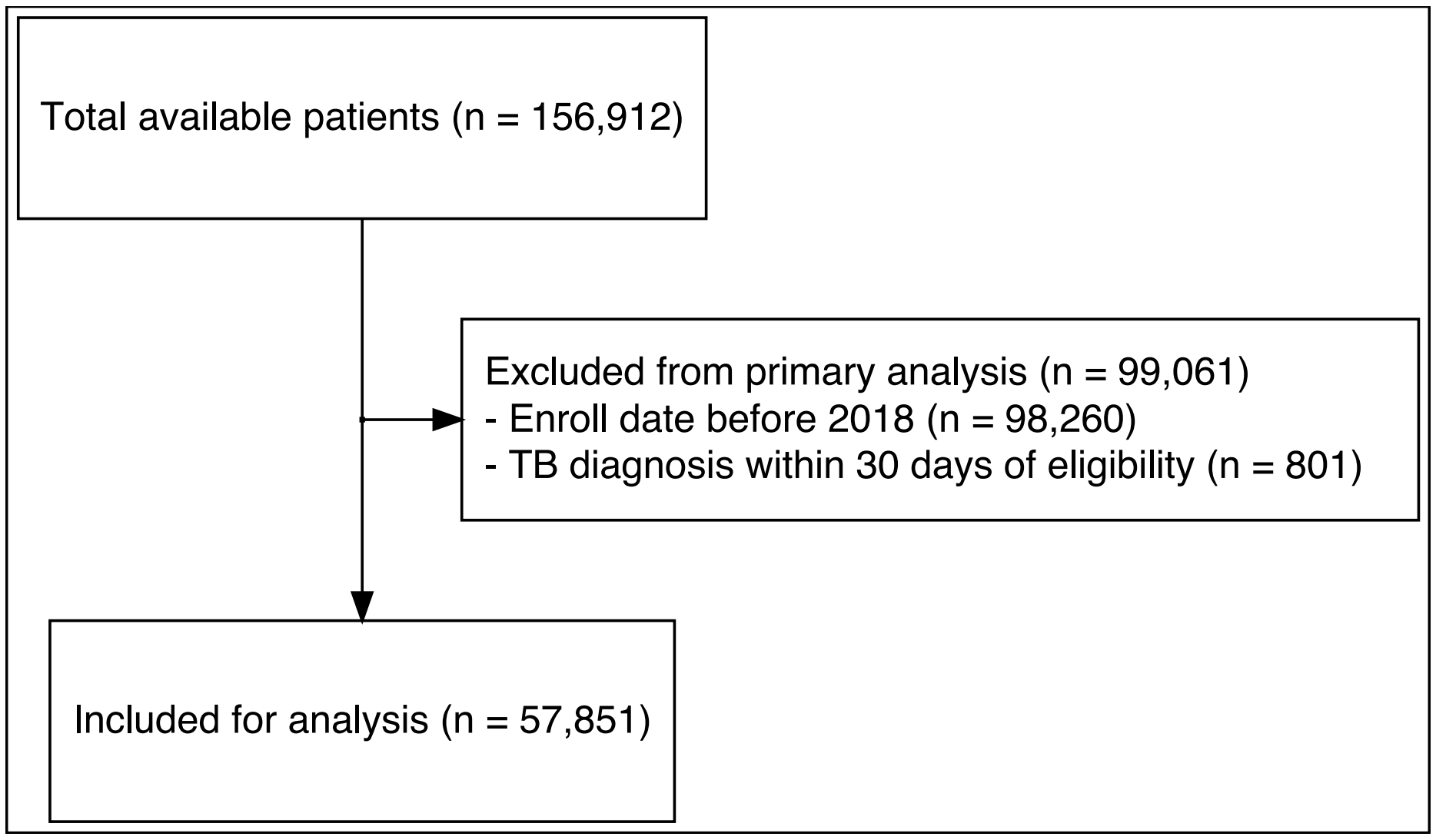

Supplemental Figure S6. Patient inclusion flow chart for Zimbabwe study cohort

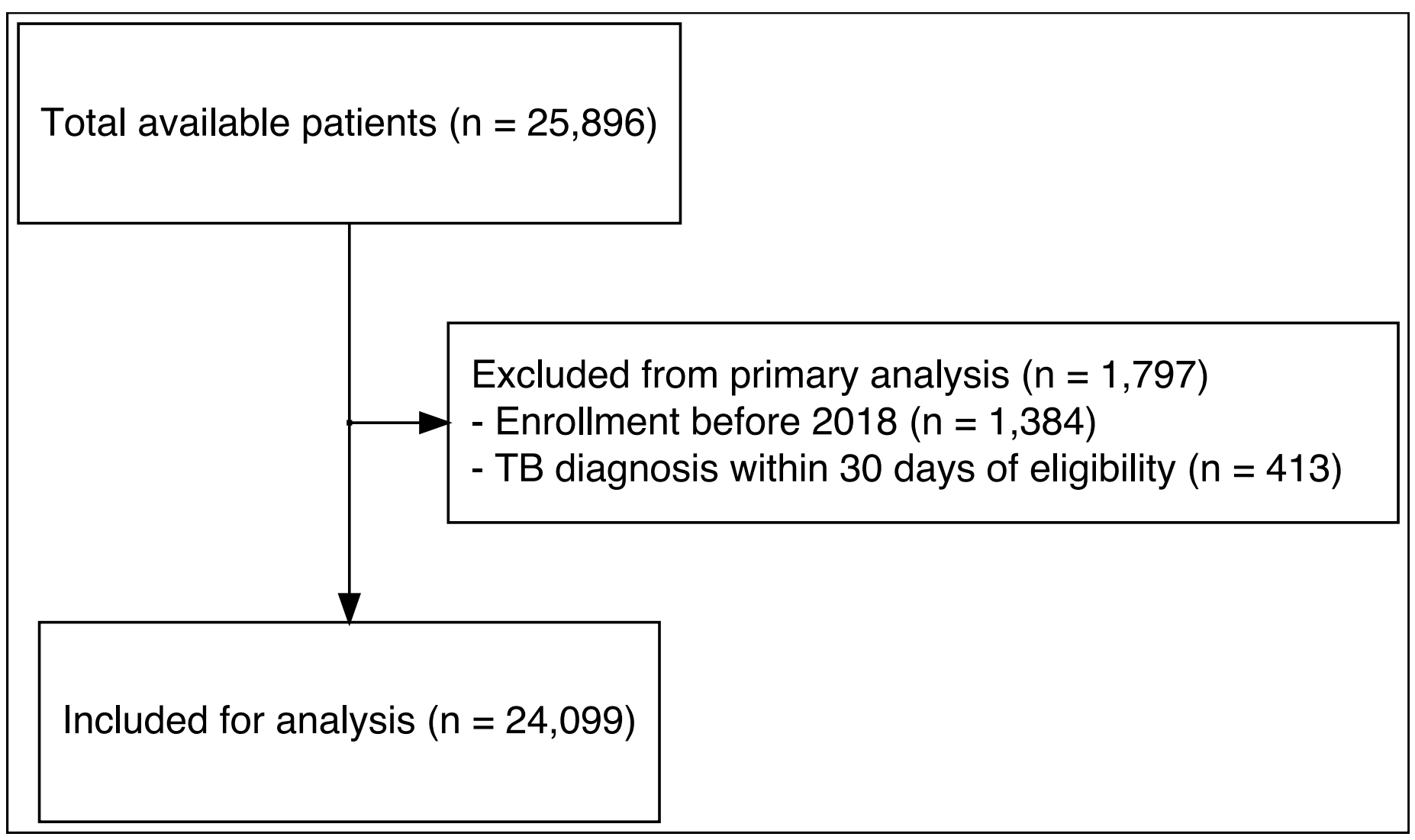

Supplemental Table S4. Demographic characteristics of Haiti study cohort by observed TPT status

Variable	Overall N = 69,936	TPT Status n (%)		
		TPT in ≤8 weeks N = 48,175	TPT in >8 weeks N = 8,522	Never TPT N = 13,239
Age				
<5 years	1,712 (2.4)	425 (0.9)	535 (6.3)	752 (5.7)
5-14 years	1,116 (1.6)	655 (1.4)	249 (2.9)	212 (1.6)
15-24 years	9,882 (14)	7,190 (15)	963 (11)	1,729 (13)
25-34 years	22,427 (32)	15,891 (33)	2,430 (29)	4,106 (31)
35-44 years	19,008 (27)	13,205 (27)	2,330 (27)	3,473 (26)
45-54 years	9,889 (14)	6,778 (14)	1,275 (15)	1,836 (14)
>55 years	5,902 (8.4)	4,031 (8.4)	740 (8.7)	1,131 (8.5)
Female	42,557 (61)	29,567 (61)	5,125 (60)	7,865 (59)
Pregnant[1]	3,954 (9.3)	3,369 (11)	267 (5.2)	318 (4.0)
Enrollment year				
2018	18,943 (27)	12,222 (25)	2,920 (34)	3,801 (29)
2019	19,334 (28)	12,980 (27)	2,757 (32)	3,597 (27)
2020	17,752 (25)	12,382 (26)	1,888 (22)	3,482 (26)
2021	13,907 (20)	10,591 (22)	957 (11)	2,359 (18)
District				
Artibonite	9,623 (14)	7,243 (15)	766 (9.0)	1,614 (12)
Centre	3,382 (4.8)	2,236 (4.6)	537 (6.3)	609 (4.6)
Grande-Anse	1,321 (1.9)	663 (1.4)	183 (2.1)	475 (3.6)
Nippes	765 (1.1)	597 (1.2)	103 (1.2)	65 (0.5)
Nord	7,015 (10)	5,306 (11)	818 (9.6)	891 (6.7)
Nord-Est	2,782 (4.0)	1,973 (4.1)	234 (2.7)	575 (4.3)
Nord-Ouest	3,632 (5.2)	2,400 (5.0)	416 (4.9)	816 (6.2)
Ouest	34,642 (50)	23,542 (49)	4,684 (55)	6,416 (48)
Sud	5,641 (8.1)	3,458 (7.2)	603 (7.1)	1,580 (12)
Sud-Est	1,133 (1.6)	757 (1.6)	178 (2.1)	198 (1.5)
Clinical Stage[2]				
Stage 1	35,665 (63)	28,197 (65)	3,788 (53)	3,680 (58)
Stage 2	13,329 (23)	10,134 (23)	1,875 (26)	1,320 (21)
Stage 3	6,502 (11)	4,227 (9.7)	1,225 (17)	1,050 (17)
Stage 4	1,436 (2.5)	881 (2.0)	256 (3.6)	299 (4.7)
Missing	13,004 (19)	4,736 (9.8)	1,378 (16)	6,890 (52)
Viral load (copies/mL)[2,3]				
<1,000	663 (73)	211 (72)	166 (66)	286 (78)
1,000 - 99,999	223 (24)	75 (25)	76 (30)	72 (20)
>=100,000	25 (2.7)	9 (3.1)	8 (3.2)	8 (2.2)
Missing	69,025 (99)	47,880 (99)	8,272 (97)	12,873 (97)
Median (IQR) days from enrollment to first viral load	43 (0, 162)	1 (0, 112)	55 (0, 182)	76 (0, 182)
ART regimen[2]				
ATV/r / LPV/r regimen	660 (1.1)	248 (0.6)	136 (1.9)	276 (2.8)
DTG regimen	53,857 (87)	39,901 (89)	6,405 (88)	7,551 (77)
EFV regimen	6,255 (10)	3,883 (8.6)	614 (8.4)	1,758 (18)
NVP regimen	170 (0.3)	47 (0.1)	29 (0.4)	94 (1.0)
Other	1,074 (1.7)	831 (1.9)	101 (1.4)	142 (1.4)
Missing	7,920 (11)	3,265 (6.8)	1,237 (15)	3,418 (26)
Weight (kg)[4]	58 (50, 65)	58 (51, 66)	56 (49, 64)	55 (48, 63)

Variable	Overall N = 69,936	TPT Status n (%) TPT in ≤8 weeks N = 48,175	TPT in >8 weeks N = 8,522	Never TPT N = 13,239
TB during follow-up	851 (1.2)	355 (0.7)	235 (2.8)	261 (2.0)
Median (IQR) days from enrollment to TB diagnosis	177 (71, 387)	300 (139, 470)	117 (52, 265)	98 (47, 247)
Death during follow-up	2,854 (4.1)	1,535 (3.2)	168 (2.0)	1,151 (8.7)
Median (IQR) days from enrollment to death	171 (58, 372)	194 (79, 392)	412 (272, 529)	107 (35, 280)
Lost to follow-up for death	33,464 (48)	21,915 (45)	2,924 (34)	8,625 (65)
Median (IQR) days from enrollment to death loss to follow-up	303 (66, 525)	338 (87, 547)	424 (243, 584)	169 (0, 411)

[1]Percent among female participants only.

[2]Percentage among participants with those measurements.

[3]First recorded measurement, not necessarily at entry into care.

[4]Presented for ages 15 and older only.

ART: antiretroviral therapy; TB: tuberculosis; TPT: tuberculosis preventive treatment

DTG: dolutegravir; EFV: efavirenz; NVP: nevirapine; ATV/r: atazanavir/ritonavir; LPV/r: lopinavir/ritonavir

Supplemental Table S5. Demographic characteristics of Kenya study cohort by observed TPT status

Variable	Overall N = 9,549	TPT Status n (%)		
		TPT in ≤8 weeks N = 6,363	TPT in >8 weeks N = 1,397	Never TPT N = 1,789
Age				
<5 years	162 (1.7)	64 (1.0)	55 (3.9)	43 (2.4)
5-14 years	225 (2.4)	94 (1.5)	49 (3.5)	82 (4.6)
15-24 years	1,511 (16)	1,061 (17)	177 (13)	273 (15)
25-34 years	3,879 (41)	2,711 (43)	522 (37)	646 (36)
35-44 years	2,460 (26)	1,627 (26)	369 (26)	464 (26)
45-54 years	1,018 (11)	651 (10)	162 (12)	205 (11)
>55 years	294 (3.1)	155 (2.4)	63 (4.5)	76 (4.2)
Female	6,555 (69)	4,317 (68)	957 (69)	1,281 (72)
Pregnant[1]	1,057 (11)	758 (12)	159 (11)	140 (7.8)
Married	4,546 (48)	3,103 (49)	640 (46)	803 (45)
Enrollment year				
2018	2,171 (23)	1,634 (26)	129 (9.2)	408 (23)
2019	3,127 (33)	2,443 (38)	192 (14)	492 (28)
2020	2,532 (27)	1,806 (28)	315 (23)	411 (23)
2021	1,719 (18)	480 (7.5)	761 (54)	478 (27)
Subcounty[2]				
Embakasi East	2,244 (24)	1,490 (23)	293 (21)	461 (26)
Embakasi West	1,614 (17)	1,052 (17)	273 (20)	289 (16)
Kamukunji	1,561 (16)	1,040 (16)	239 (17)	282 (16)
Kasarani	2,027 (21)	1,357 (21)	283 (20)	387 (22)
Ruaraka	664 (7.0)	489 (7.7)	72 (5.2)	103 (5.8)
Starehe	1,428 (15)	934 (15)	235 (17)	259 (15)
Missing	11 (0.1)	1 (<0.1)	2 (0.1)	8 (0.4)
Education				
Primary	4,451 (47)	3,005 (47)	586 (42)	860 (48)
Secondary	3,581 (38)	2,468 (39)	542 (39)	571 (32)
Tertiary	1,030 (11)	671 (11)	164 (12)	195 (11)
None/Unknown	487 (5.1)	219 (3.4)	105 (7.5)	163 (9.1)
Religion				
Catholic	4,633 (49)	3,114 (49)	673 (48)	846 (47)
Muslim	209 (2.2)	119 (1.9)	37 (2.6)	53 (3.0)
Other Christian	4,001 (42)	2,707 (43)	557 (40)	737 (41)
Other/Unknown	706 (7.4)	423 (6.6)	130 (9.3)	153 (8.6)
Clinical stage[2]				
Stage 1	4,846 (51)	3,315 (52)	676 (48)	855 (48)
Stage 2	3,246 (34)	2,257 (35)	445 (32)	544 (30)
Stage 3	1,342 (14)	744 (12)	254 (18)	344 (19)
Stage 4	115 (1.2)	47 (0.7)	22 (1.6)	46 (2.6)
CD4 count (cells/mm3)[2,3]				
0-199	1,431 (20)	962 (20)	269 (22)	200 (19)
200-349	1,292 (18)	948 (20)	218 (18)	126 (12)
350-499	1,284 (18)	931 (20)	191 (16)	162 (15)
>500	2,989 (43)	1,892 (40)	527 (44)	570 (54)
Missing	2,553 (27)	1,630 (26)	192 (14)	731 (41)
Median (IQR) days from enrollment to first CD4 count	2 (1, 13)	2 (1, 11)	2 (1, 20)	1 (0, 11)

Variable	Overall N = 9,549	TPT Status n (%)		
		TPT in ≤8 weeks N = 6,363	TPT in >8 weeks N = 1,397	Never TPT N = 1,789
Viral load (copies/mL)[2,3]				
<1,000	5,920 (81)	3,954 (80)	1,090 (85)	876 (82)
1,000 - 99,999	970 (13)	703 (14)	132 (10)	135 (13)
>=100,000	405 (5.6)	283 (5.7)	66 (5.1)	56 (5.2)
Missing	2,254 (24)	1,423 (22)	109 (7.8)	722 (40)
Median (IQR) days from enrollment to first viral load	183 (91, 241)	184 (156, 219)	295 (169, 443)	4 (0, 97)
ART regimen[2]				
ATV/r / LPV/r regimen	311 (3.4)	125 (2.1)	42 (3.2)	144 (8.5)
DTG regimen	7,004 (77)	4,587 (76)	1,243 (94)	1,174 (69)
EFV regimen	1,756 (19)	1,346 (22)	37 (2.8)	373 (22)
NVP regimen	23 (0.3)	9 (0.1)	1 (<0.1)	13 (0.8)
Missing	455 (4.8)	296 (4.7)	74 (5.3)	85 (4.8)
Body Mass Index (BMI) category[2]				
Underweight	839 (11)	454 (8.7)	127 (11)	258 (17)
Normal Weight	4,057 (52)	2,726 (52)	584 (51)	747 (50)
Overweight	1,914 (24)	1,326 (25)	264 (23)	324 (22)
Obese	1,037 (13)	713 (14)	171 (15)	153 (10)
Missing	1,702 (18)	1,144 (18)	251 (18)	307 (17)
Known previous TB diagnosis prior to enrollment	30 (0.3)	17 (0.3)	9 (0.6)	4 (0.2)
TB during follow-up	178 (1.9)	78 (1.2)	61 (4.4)	39 (2.2)
Median (IQR) days from enrollment to TB diagnosis	135 (64, 355)	213 (87, 469)	120 (49, 306)	89 (55, 217)
Death during follow-up	324 (3.4)	144 (2.3)	10 (0.7)	170 (9.5)
Median (IQR) days from enrollment to death	126 (49, 340)	219 (106, 420)	521 (298, 689)	71 (29, 151)
Lost to follow-up for TB	4,279 (45)	2,824 (44)	326 (23)	1,129 (63)
Median (IQR) days from enrollment to TB loss to follow-up	133 (25, 339)	144 (33, 351)	346 (195, 526)	61 (2, 211)
Lost to follow-up for death	3,167 (33)	2,092 (33)	198 (14)	877 (49)
Median (IQR) days from enrollment to death loss to follow-up	144 (28, 365)	148 (32, 371)	443 (266, 582)	85 (7, 270)

[1]Percent among female participants only.

[2]Percentage among participants with those measurements.

[3]First recorded measurement, not necessarily at entry into care.

ART: antiretroviral therapy; TB: tuberculosis; TPT: tuberculosis preventive treatment

DTG: dolutegravir; EFV: efavirenz; NVP: nevirapine; ATV/r: atazanavir/ritonavir; LPV/r: lopinavir/ritonavir

Supplemental Table S6. Demographic characteristics of Nigeria study cohort by observed TPT status

Variable	Overall N = 8,053	TPT Status n (%) TPT in ≤8 weeks N = 3,677	TPT in >8 weeks N = 3,909	Never TPT N = 467
Age				
<5 years	56 (0.7)	6 (0.2)	42 (1.1)	8 (1.7)
5-14 years	69 (0.9)	14 (0.4)	41 (1.0)	14 (3.0)
15-24 years	1,997 (25)	970 (26)	939 (24)	88 (19)
25-34 years	3,753 (47)	1,829 (50)	1,748 (45)	176 (38)
35-44 years	1,599 (20)	670 (18)	818 (21)	111 (24)
45-54 years	424 (5.3)	155 (4.2)	223 (5.7)	46 (9.9)
>55 years	155 (1.9)	33 (0.9)	98 (2.5)	24 (5.1)
Female	4,052 (50)	1,840 (50)	1,912 (49)	300 (64)
Pregnant[1]	61 (1.5)	19 (1.0)	34 (1.8)	8 (2.7)
Breastfeeding[1]	22 (0.5)	6 (0.3)	15 (0.8)	1 (0.3)
Enrollment year				
2018	657 (8.2)	30 (0.8)	506 (13)	121 (26)
2019	872 (11)	79 (2.1)	695 (18)	98 (21)
2020	3,921 (49)	1,608 (44)	2,154 (55)	159 (34)
2021	2,603 (32)	1,960 (53)	554 (14)	89 (19)
State				
Benue	969 (12)	103 (2.8)	747 (19)	119 (25)
Delta	1,066 (13)	489 (13)	525 (13)	52 (11)
Enugu	722 (9.0)	428 (12)	249 (6.4)	45 (9.6)
Federal Capital Territory	1,087 (13)	600 (16)	465 (12)	22 (4.7)
Kaduna	236 (2.9)	40 (1.1)	144 (3.7)	52 (11)
Lagos	875 (11)	329 (8.9)	477 (12)	69 (15)
Nasarawa	907 (11)	559 (15)	334 (8.5)	14 (3.0)
Plateau	136 (1.7)	6 (0.2)	107 (2.7)	23 (4.9)
Rivers	2,055 (26)	1,123 (31)	861 (22)	71 (15)
Clinical Stage[2]				
Stage 1	6,791 (96)	3,439 (98)	3,065 (93)	287 (91)
Stage 2	228 (3.2)	38 (1.1)	176 (5.3)	14 (4.4)
Stage 3	69 (1.0)	15 (0.4)	42 (1.3)	12 (3.8)
Stage 4	11 (0.2)	2 (<0.1)	7 (0.2)	2 (0.6)
Missing	954 (12)	183 (5.0)	619 (16)	152 (33)
CD4 count (cells/mm3)[2,3]				
0-199	92 (31)	11 (31)	68 (31)	13 (32)
200-349	70 (24)	9 (26)	51 (23)	10 (24)
350-499	52 (18)	6 (17)	40 (18)	6 (15)
>500	83 (28)	9 (26)	62 (28)	12 (29)
Missing	7,756 (96)	3,642 (99)	3,688 (94)	426 (91)
Median (IQR) days from enrollment to first CD4 count	0 (-5, 61)	0 (-118, 59)	0 (-1, 67)	0 (-7, 29)
Viral load (copies/mL)[2,3]				
<1,000	2,414 (93)	723 (96)	1,495 (93)	196 (88)
1,000 - 99,999	111 (4.3)	25 (3.3)	72 (4.5)	14 (6.3)
>=100,000	61 (2.4)	6 (0.8)	42 (2.6)	13 (5.8)
Missing	5,467 (68)	2,923 (79)	2,300 (59)	244 (52)
Median (IQR) days from enrollment to first viral load	488 (198, 869)	517 (196, 883)	471 (198, 855)	462 (224, 854)

Variable	Overall N = 8,053	TPT Status n (%)		
		TPT in ≤8 weeks N = 3,677	TPT in >8 weeks N = 3,909	Never TPT N = 467
ART regimen[2]				
ATV/r / LPV/r regimen	42 (0.6)	5 (0.1)	28 (0.8)	9 (2.2)
DTG regimen	6,740 (90)	3,458 (98)	3,013 (84)	269 (67)
EFV regimen	563 (7.5)	38 (1.1)	433 (12)	92 (23)
NVP regimen	139 (1.9)	9 (0.3)	97 (2.7)	33 (8.2)
Other	6 (<0.1)	4 (0.1)	2 (<0.1)	0 (0)
Missing	563 (7.0)	163 (4.4)	336 (8.6)	64 (14)
High alcohol use	1,469 (18)	989 (27)	455 (12)	25 (5.4)
TB during follow-up	19 (0.2)	3 (<0.1)	14 (0.4)	2 (0.4)
Median (IQR) days from enrollment to TB diagnosis	253 (91, 466)	38 (37, 85)	268 (207, 466)	420 (371, 468)
Death during follow-up	28 (0.3)	10 (0.3)	8 (0.2)	10 (2.1)
Median (IQR) days from enrollment to death	312 (109, 549)	410 (99, 609)	501 (255, 569)	159 (107, 412)
Lost to follow-up for TB	1,646 (20)	836 (23)	667 (17)	143 (31)
Median (IQR) days from enrollment to TB loss to follow-up	265 (80, 592)	439 (80, 629)	194 (81, 479)	206 (70, 406)
Lost to follow-up for death	1,925 (24)	1,020 (28)	750 (19)	155 (33)
Median (IQR) days from enrollment to death loss to follow-up	339 (87, 617)	503 (107, 646)	211 (83, 515)	206 (36, 428)

[1]Percent among female participants only.

[2]Percentage among participants with those measurements.

[3]First recorded measurement, not necessarily at entry into care.

ART: antiretroviral therapy; TB: tuberculosis; TPT: tuberculosis preventive treatment

DTG: dolutegravir; EFV: efavirenz; NVP: nevirapine; ATV/r: atazanavir/ritonavir; LPV/r: lopinavir/ritonavir

Supplemental Table S7. Demographic characteristics of Uganda study cohort by observed TPT status

Variable	Overall N = 65,936	TPT Status		
		TPT in ≤8 weeks N = 7,174	TPT in >8 weeks N = 29,475	Never TPT N = 29,287
Age				
<5 years	1,714 (2.6)	93 (1.3)	804 (2.7)	817 (2.8)
5-14 years	1,194 (1.8)	123 (1.7)	618 (2.1)	453 (1.5)
15-24 years	15,920 (24)	1,651 (23)	6,463 (22)	7,806 (27)
25-34 years	25,288 (38)	2,916 (41)	11,166 (38)	11,206 (38)
35-44 years	13,749 (21)	1,552 (22)	6,506 (22)	5,691 (19)
45-54 years	5,651 (8.6)	605 (8.4)	2,780 (9.4)	2,266 (7.7)
>55 years	2,420 (3.7)	234 (3.3)	1,138 (3.9)	1,048 (3.6)
Female	42,526 (64)	4,678 (65)	18,804 (64)	19,044 (65)
Pregnant[1]	6,279 (15)	806 (17)	2,817 (15)	2,656 (14)
Breastfeeding[1]	561 (1.3)	54 (1.2)	264 (1.4)	243 (1.3)
Enrollment year				
2018	21,512 (33)	856 (12)	9,006 (31)	11,650 (40)
2019	15,595 (24)	2,732 (38)	5,809 (20)	7,054 (24)
2020	11,219 (17)	1,716 (24)	5,119 (17)	4,384 (15)
2021	9,332 (14)	1,146 (16)	5,014 (17)	3,172 (11)
2022	8,278 (13)	724 (10)	4,527 (15)	3,027 (10)
Region				
Acholi	7,612 (12)	867 (12)	3,329 (11)	3,416 (12)
Ankole	5,627 (8.5)	639 (8.9)	2,297 (7.8)	2,691 (9.2)
Bugisu	1,431 (2.2)	91 (1.3)	783 (2.7)	557 (1.9)
Bukedi	3,904 (5.9)	110 (1.5)	1,715 (5.8)	2,079 (7.1)
Bunyoro	1,728 (2.6)	175 (2.4)	623 (2.1)	930 (3.2)
Busoga	3,734 (5.7)	464 (6.5)	2,392 (8.1)	878 (3.0)
Kampala	14,280 (22)	2,027 (28)	5,965 (20)	6,288 (21)
Karamoja	756 (1.1)	117 (1.6)	366 (1.2)	273 (0.9)
Kigezi	2,169 (3.3)	245 (3.4)	971 (3.3)	953 (3.3)
Lango	965 (1.5)	53 (0.7)	288 (1.0)	624 (2.1)
North Central	6,272 (9.5)	545 (7.6)	3,122 (11)	2,605 (8.9)
South Central	4,365 (6.6)	202 (2.8)	2,311 (7.8)	1,852 (6.3)
Teso	1,852 (2.8)	177 (2.5)	984 (3.3)	691 (2.4)
Tooro	8,088 (12)	1,068 (15)	2,755 (9.3)	4,265 (15)
West Nile	3,153 (4.8)	394 (5.5)	1,574 (5.3)	1,185 (4.0)
Clinical Stage[2]				
Stage 1	43,829 (77)	5,055 (79)	19,952 (77)	18,822 (77)
Stage 2	9,595 (17)	1,092 (17)	4,579 (18)	3,924 (16)
Stage 3	2,539 (4.5)	191 (3.0)	1,113 (4.3)	1,235 (5.1)
Stage 4	775 (1.4)	42 (0.7)	279 (1.1)	454 (1.9)
Missing	9,198 (14)	794 (11)	3,552 (12)	4,852 (17)
CD4 count (cells/mm3)[2,3]				
0-199	4,754 (23)	570 (21)	2,438 (24)	1,746 (24)
200-349	3,893 (19)	512 (19)	2,071 (20)	1,310 (18)
350-499	3,762 (19)	536 (20)	1,910 (19)	1,316 (18)
>500	7,872 (39)	1,058 (40)	3,843 (37)	2,971 (40)
Missing	45,655 (69)	4,498 (63)	19,213 (65)	21,944 (75)
Median (IQR) days from enrollment to first CD4 count	0.00 (0.00, 0.00)	0.00 (0.00, 0.00)	0.00 (0.00, 0.00)	0.00 (0.00, 0.00)

Variable	Overall<br>N = 65,936	TPT Status		
		TPT in<br>≤8 weeks<br>N = 7,174	TPT in<br>>8 weeks<br>N = 29,475	Never TPT<br>N = 29,287
Viral load (copies/mL)[2,3]				
<1,000	35,909 (92)	5,326 (93)	24,304 (92)	6,279 (89)
1,000 - 99,999	2,484 (6.4)	308 (5.4)	1,570 (6.0)	606 (8.6)
>=100,000	698 (1.8)	75 (1.3)	425 (1.6)	198 (2.8)
Missing	26,845 (41)	1,465 (20)	3,176 (11)	22,204 (76)
Median (IQR) days from enrollment to first viral load	202 (174, 294)	190 (168, 244)	204 (175, 302)	204 (170, 313)
Body Mass Index (BMI) category[2]				
Underweight	5,388 (15)	668 (13)	3,395 (15)	1,325 (16)
Normal Weight	20,993 (59)	3,103 (60)	13,224 (59)	4,666 (58)
Overweight	6,262 (18)	953 (18)	3,886 (17)	1,423 (18)
Obese	3,055 (8.6)	487 (9.3)	1,908 (8.5)	660 (8.2)
Missing	30,238 (46)	1,963 (27)	7,062 (24)	21,213 (72)
ART regimen[2]				
ATV/r / LPV/r regimen	1,270 (2.0)	96 (1.4)	632 (2.2)	542 (1.9)
DTG regimen	26,942 (42)	3,306 (47)	13,836 (48)	9,800 (34)
EFV regimen	31,497 (49)	2,828 (41)	12,567 (43)	16,102 (56)
NVP regimen	756 (1.2)	26 (0.4)	340 (1.2)	390 (1.4)
Other	4,167 (6.4)	716 (10)	1,672 (5.8)	1,779 (6.2)
Missing	1,304 (2.0)	202 (2.8)	428 (1.5)	674 (2.3)
TB during follow-up	775 (1.2)	57 (0.8)	453 (1.5)	265 (0.9)
Median (IQR) days from enrollment to TB diagnosis	183 (76, 399)	260 (154, 454)	198 (84, 405)	133 (59, 358)
Death during follow-up	2,333 (3.5)	66 (0.9)	148 (0.5)	2,119 (7.2)
Median (IQR) days from enrollment to death	97 (28, 281)	324 (142, 484)	417 (270, 564)	80 (25, 232)
Lost to follow-up for TB	33,671 (51)	2,296 (32)	7,121 (24)	24,254 (83)
Median (IQR) days from enrollment to TB loss to follow-up	143 (14, 412)	329 (153, 544)	507 (309, 618)	63 (0, 236)
Lost to follow-up for death	31,654 (48)	2,278 (32)	7,086 (24)	22,290 (76)
Median (IQR) days from enrollment to death loss to follow-up	118 (0, 406)	276 (120, 534)	504 (297, 617)	41 (0, 198)

[1]Percent among female participants only.
[2]Percentage among participants with those measurements.
[3]First recorded measurement, not necessarily at entry into care.
ART: antiretroviral therapy; TB: tuberculosis; TPT: tuberculosis preventive treatment
DTG: dolutegravir; EFV: efavirenz; NVP: nevirapine; ATV/r: atazanavir/ritonavir; LPV/r: lopinavir/ritonavir

Supplemental Table S8. Demographic characteristics of Ukraine study cohort by observed TPT status

Variable	Overall N = 57,851	TPT Status		
		TPT in ≤8 weeks N = 25,650	TPT in >8 weeks N = 8,122	Never TPT N = 24,079
Age				
<5 years	253 (0.4)	9 (<0.1)	14 (0.2)	230 (1.0)
5-14 years	184 (0.3)	25 (<0.1)	27 (0.3)	132 (0.5)
15-24 years	2,748 (4.8)	1,117 (4.4)	382 (4.7)	1,249 (5.2)
25-34 years	17,276 (30)	7,537 (29)	2,394 (29)	7,345 (31)
35-44 years	23,572 (41)	10,644 (41)	3,211 (40)	9,717 (40)
45-54 years	10,139 (18)	4,617 (18)	1,519 (19)	4,003 (17)
>55 years	3,679 (6.4)	1,701 (6.6)	575 (7.1)	1,403 (5.8)
Female	22,414 (39)	9,869 (38)	3,414 (42)	9,131 (38)
Male	35,437 (61)	15,781 (62)	4,708 (58)	14,948 (62)
Enrollment year				
2018	13,945 (24)	3,326 (13)	3,422 (42)	7,197 (30)
2019	14,742 (25)	4,873 (19)	2,707 (33)	7,162 (30)
2020	14,889 (26)	8,043 (31)	1,537 (19)	5,309 (22)
2021	14,275 (25)	9,408 (37)	456 (5.6)	4,411 (18)
CD4 count (cells/mm3)[1,2]				
0-199	17,325 (32)	7,824 (33)	3,017 (38)	6,484 (30)
200-349	12,265 (23)	5,747 (24)	1,916 (24)	4,602 (21)
350-499	10,182 (19)	4,586 (19)	1,392 (17)	4,204 (19)
>500	14,046 (26)	5,830 (24)	1,646 (21)	6,570 (30)
Missing	4,033 (7.0)	1,663 (6.5)	151 (1.9)	2,219 (9.2)
Median (IQR) days from enrollment to first CD4 count	0 (-5, 6)	1 (-1, 4)	1 (-7, 14)	0 (-8, 6)
Viral load (copies/mL)[1,2]				
<1,000	15,957 (32)	6,463 (29)	2,193 (28)	7,301 (36)
1,000 - 99,999	21,588 (43)	9,856 (45)	3,406 (43)	8,326 (41)
>=100,000	12,723 (25)	5,599 (26)	2,231 (28)	4,893 (24)
Missing	7,583 (13)	3,732 (15)	292 (3.6)	3,559 (15)
Median (IQR) days from enrollment to first viral load	4 (-1, 29)	5 (0, 19)	5 (-3, 57)	3 (-6, 42)
ART regimen[1]				
ATV/r / LPV/r regimen	4,885 (8.4)	1,493 (5.8)	872 (11)	2,520 (10)
DTG regimen	32,633 (56)	16,883 (66)	4,034 (50)	11,716 (49)
EFV regimen	20,009 (35)	7,230 (28)	3,170 (39)	9,609 (40)
NVP regimen	193 (0.3)	19 (<0.1)	31 (0.4)	143 (0.6)
Other	93 (0.2)	21 (<0.1)	15 (0.2)	57 (0.2)
Missing	38 (<0.1)	4 (<0.1)	0 (0)	34 (0.1)
High alcohol use	3,512 (6.1)	1,894 (7.4)	403 (5.0)	1,215 (5.0)
History of imprisonment	4,040 (7.0)	661 (2.6)	473 (5.8)	2,906 (12)
TB during follow-up	1,741 (3.0)	300 (1.2)	243 (3.0)	1,198 (5.0)
Median (IQR) days from enrollment to TB diagnosis	264 (120, 435)	271 (133, 432)	314 (151, 462)	255 (110, 425)
Death during follow-up	3,607 (6.2)	1,220 (4.8)	228 (2.8)	2,159 (9.0)
Median (IQR) days from enrollment to death	166 (60, 403)	160 (66, 352)	458 (300, 601)	148 (52, 392)
Lost to follow-up for TB	11,315 (20)	4,525 (18)	1,306 (16)	5,484 (23)
Median (IQR) days from	287 (165, 453)	258 (155, 396)	385 (214, 551)	301 (166, 470)

Variable	Overall N = 57,851	TPT Status		
		TPT in ≤8 weeks N = 25,650	TPT in >8 weeks N = 8,122	Never TPT N = 24,079
enrollment to TB loss to follow-up				
Lost to follow-up for death	11,015 (19)	4,438 (17)	1,285 (16)	5,292 (22)
Median (IQR) days from enrollment to death loss to follow-up	287 (166, 454)	258 (155, 396)	384 (213, 548)	304 (168, 472)

[1]Percentage among participants with those measurements.

[2]First recorded measurement, not necessarily at entry into care.

ART: antiretroviral therapy; TB: tuberculosis; TPT: tuberculosis preventive treatment

DTG: dolutegravir; EFV: efavirenz; NVP: nevirapine; ATV/r: atazanavir/ritonavir; LPV/r: lopinavir/ritonavir

Supplemental Table S9. Demographic characteristics of Zimbabwe study cohort by observed TPT status

Variable	Overall N = 24,099	TPT Status: TPT in ≤8 weeks N = 6,214	TPT in >8 weeks N = 9,054	Never TPT N = 8,831
Age				
<5 years	421 (1.7)	38 (0.6)	118 (1.3)	265 (3.0)
5-14 years	468 (1.9)	72 (1.2)	201 (2.2)	195 (2.2)
15-24 years	4,754 (20)	1,331 (21)	1,511 (17)	1,912 (22)
25-34 years	8,245 (34)	2,238 (36)	2,982 (33)	3,025 (34)
35-44 years	6,411 (27)	1,650 (27)	2,591 (29)	2,170 (25)
45-54 years	2,741 (11)	669 (11)	1,208 (13)	864 (9.8)
>55 years	1,059 (4.4)	216 (3.5)	443 (4.9)	400 (4.5)
Female	15,478 (64)	4,069 (65)	5,725 (63)	5,684 (64)
Pregnant[1]	656 (4.2)	300 (7.4)	163 (2.8)	193 (3.4)
Breastfeeding[1]	537 (3.5)	118 (2.9)	204 (3.6)	215 (3.8)
Enrollment year				
2018	3,277 (14)	6 (<0.1)	1,821 (20)	1,450 (16)
2019	7,555 (31)	515 (8.3)	3,921 (43)	3,119 (35)
2020	4,780 (20)	1,409 (23)	1,675 (19)	1,696 (19)
2021	4,412 (18)	1,988 (32)	1,021 (11)	1,403 (16)
2022	4,075 (17)	2,296 (37)	616 (6.8)	1,163 (13)
District				
Chegutu	2,376 (9.9)	187 (3.0)	1,233 (14)	956 (11)
Chitungwiza	2,921 (12)	617 (9.9)	776 (8.6)	1,528 (17)
Goromonzi	1,949 (8.1)	505 (8.1)	884 (9.8)	560 (6.3)
ha North Western District	1,826 (7.6)	381 (6.1)	846 (9.3)	599 (6.8)
ha South Western District	1,388 (5.8)	283 (4.6)	608 (6.7)	497 (5.6)
ha Southern District	1,568 (6.5)	915 (15)	101 (1.1)	552 (6.3)
ha West South West District	4,373 (18)	1,518 (24)	1,812 (20)	1,043 (12)
Makonde	799 (3.3)	172 (2.8)	322 (3.6)	305 (3.5)
Marondera	1,131 (4.7)	361 (5.8)	356 (3.9)	414 (4.7)
Mazowe	1,016 (4.2)	536 (8.6)	261 (2.9)	219 (2.5)
Nkulumane	1,725 (7.2)	158 (2.5)	538 (5.9)	1,029 (12)
Sanyati	1,900 (7.9)	373 (6.0)	896 (9.9)	631 (7.1)
Tsholotsho	1,127 (4.7)	208 (3.3)	421 (4.6)	498 (5.6)
Clinical Stage[2]				
Stage 1	13,146 (63)	4,091 (72)	4,839 (58)	4,216 (61)
Stage 2	5,092 (24)	1,169 (21)	2,295 (27)	1,628 (23)
Stage 3	2,558 (12)	401 (7.1)	1,140 (14)	1,017 (15)
Stage 4	176 (0.8)	26 (0.5)	75 (0.9)	75 (1.1)
Missing	3,127 (13)	527 (8.5)	705 (7.8)	1,895 (21)
CD4 count (cells/mm3)[2,3]				
0-199	206 (33)	25 (44)	86 (29)	95 (34)
200-349	154 (24)	11 (19)	79 (27)	64 (23)
350-499	103 (16)	8 (14)	51 (17)	44 (16)
>500	166 (26)	13 (23)	76 (26)	77 (28)
Missing	23,470 (97)	6,157 (99)	8,762 (97)	8,551 (97)
Median (IQR) days from enrollment to first CD4 count	0 (-76, 0)	0 (0, 1)	-58 (-89, 1)	-62 (-90, 0)
Viral load (copies/mL)[2,3]				
<1,000	13,393 (94)	3,912 (96)	6,837 (95)	2,644 (91)

Variable	Overall N = 24,099	TPT Status		
		TPT in ≤8 weeks N = 6,214	TPT in >8 weeks N = 9,054	Never TPT N = 8,831
1,000 - 99,999	674 (4.7)	134 (3.3)	321 (4.4)	219 (7.5)
>=100,000	136 (1.0)	24 (0.6)	64 (0.9)	48 (1.6)
Missing	9,896 (41)	2,144 (35)	1,832 (20)	5,920 (67)
Median (IQR) days from enrollment to first viral load	428 (201, 872)	218 (180, 385)	709 (354, 1,024)	471 (203, 898)
ART regimen[2]				
ATV/r / LPV/r regimen	398 (1.7)	21 (0.3)	158 (1.7)	219 (2.5)
DTG regimen	11,738 (49)	4,924 (79)	3,670 (41)	3,144 (36)
EFV regimen	9,070 (38)	721 (12)	4,293 (47)	4,056 (46)
NVP regimen	160 (0.7)	12 (0.2)	65 (0.7)	83 (0.9)
Other	2,733 (11)	536 (8.6)	868 (9.6)	1,329 (15)
TB during follow-up	235 (1.0)	36 (0.6)	81 (0.9)	118 (1.3)
Median (IQR) days from enrollment to TB diagnosis	176 (60, 368)	124 (56, 312)	183 (71, 372)	182 (59, 368)
Death during follow-up	386 (1.6)	98 (1.6)	37 (0.4)	251 (2.8)
Median (IQR) days from enrollment to death	120 (15, 346)	92 (7, 219)	403 (157, 564)	102 (15, 318)
Lost to follow-up for TB	9,668 (40)	3,303 (53)	1,267 (14)	5,098 (58)
Median (IQR) days from enrollment to TB loss to follow-up	241 (31, 505)	272 (84, 537)	484 (303, 624)	162 (7, 406)
Lost to follow-up for death	9,139 (38)	3,135 (50)	1,114 (12)	4,890 (55)
Median (IQR) days from enrollment to death loss to follow-up	273 (71, 512)	354 (178, 545)	509 (363, 630)	158 (4, 404)

[1]Percent among female participants only.

[2]Percentage among participants with those measurements.

[3]First recorded measurement, not necessarily at entry into care.

ART: antiretroviral therapy; TB: tuberculosis; TPT: tuberculosis preventive treatment

DTG: dolutegravir; EFV: efavirenz; NVP: nevirapine; ATV/r: atazanavir/ritonavir; LPV/r: lopinavir/ritonavir

Supplemental Table S10. Person time contributions for estimation of TPT effectiveness across countries

TB outcome

Country	Data for estimating incidence of TB under 8-week grace period for TPT initiation			Data for estimating incidence of TB under control (no TPT) condition		
	n[1]	Person-weeks Total	Person-weeks Median (IQR)[2]	n[1]	Person-weeks Total	Person-weeks Median (IQR)[2]
Haiti	63,578	3,799,846	43 (19, 71)	25,244	1,065,571	30 (11, 59)
Kenya	8,814	473,217	43 (18, 72)	4,177	157,828	31 (11, 62)
Nigeria	7,708	367,576	45 (17, 73)	4,393	249,001	34 (14, 61)
Uganda	56,273	976,123	15 (5, 56)	54,099	2,254,362	29 (12, 55)
Ukraine	57,665	2,538,757	43 (16, 73)	38,535	2,481,268	43 (19, 71)
Zimbabwe	22,039	565,485	30 (7, 63)	17,554	1,218,202	42 (19, 70)

All-cause mortality outcome

Country	Data for estimating all-cause mortality under 8-week grace period for TPT initiation			Data for estimating all-cause mortality under control (no TPT) condition		
	n[1]	Person-weeks Total	Person-weeks Median (IQR)[2]	n[1]	Person-weeks Total	Person-weeks Median (IQR)[2]
Haiti	63,578	3,812,856	44 (19, 71)	25,244	1,086,345	31 (11, 59)
Kenya	9,007	523,922	45 (19, 74)	4,282	174,420	33 (12, 64)
Nigeria	7,714	368,072	45 (17, 73)	4,396	249,544	34 (15, 61)
Uganda	56,974	984,096	15 (5, 56)	54,800	2,293,082	29 (12, 56)
Ukraine	57,673	2,553,399	43 (16, 73)	38,542	2,543,061	44 (20, 72)
Zimbabwe	22,560	596,997	30 (7, 63)	17,733	1,244,101	42 (20, 70)

[1] Number of unique patients that contribute at least one person-week of follow up time to estimation of incidence of the given endpoint

[2] Among patients that contribute at least one person-week of follow up time

TB: tuberculosis; TPT: tuberculosis preventive treatment

Supplemental Figure S7. Two-year cumulative incidence (95% CI) of TB under each treatment scenario by country comparing TPT initiation within 2 weeks of enrollment into care to never initiating TPT

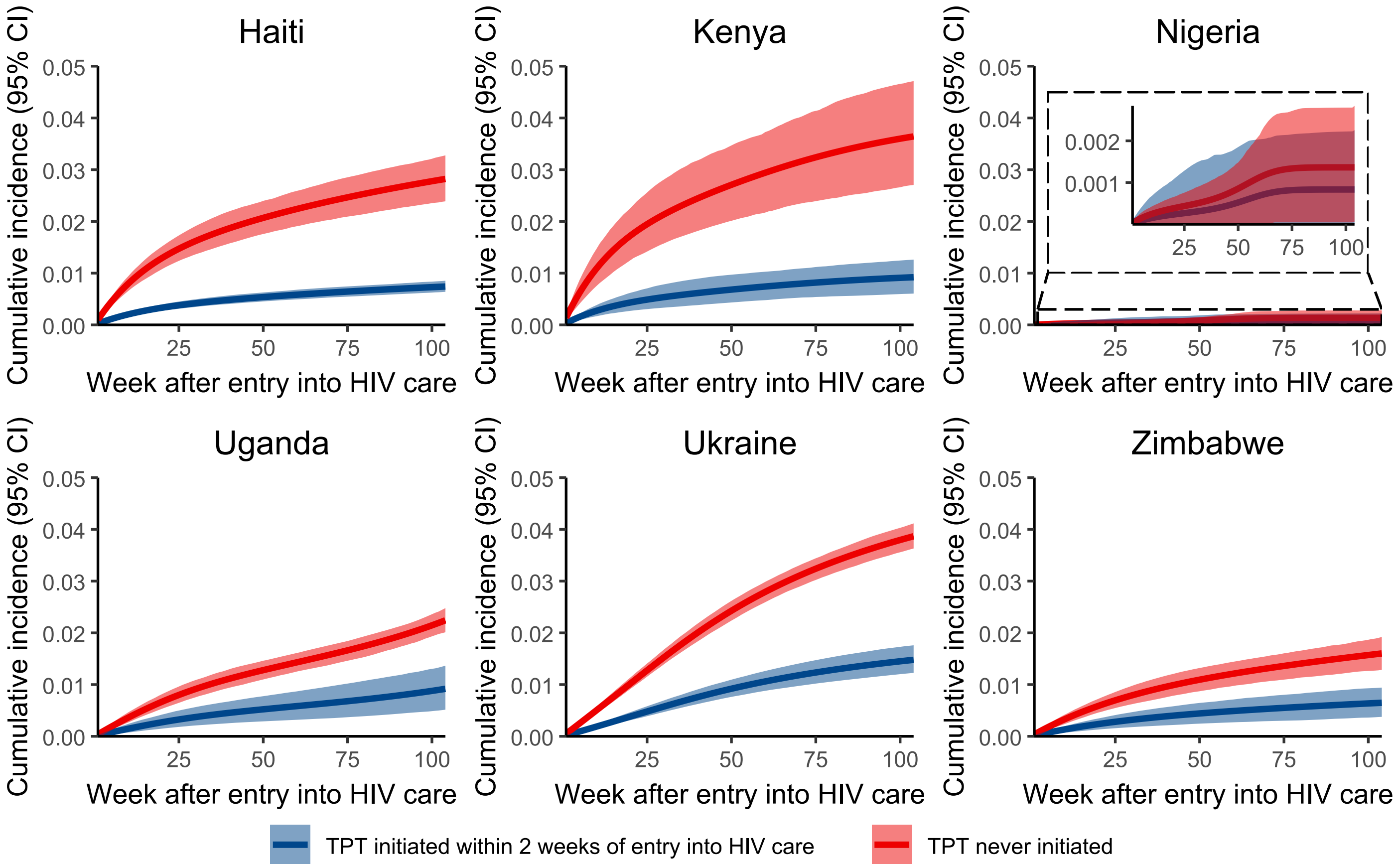

Supplemental Figure 8. Two-year cumulative incidence (95% CI) of all-cause mortality under each treatment scenario by country comparing TPT initiation within 2 weeks of enrollment into care to never initiating TPT

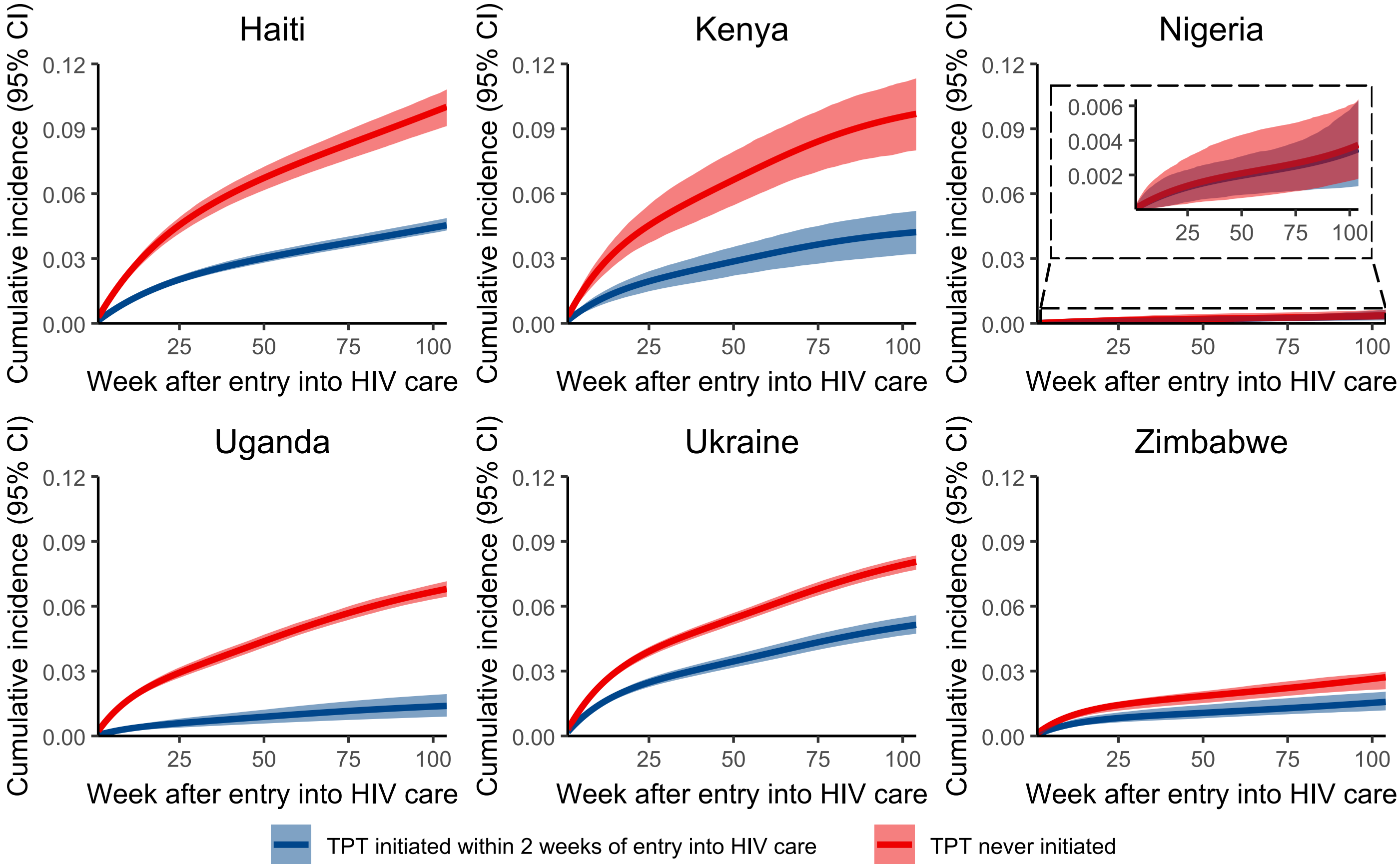

Supplemental Table S12. Adjusted hazard ratios for tuberculosis diagnosis comparing TPT initiation within 2 weeks of enrollment into care to never initiating TPT, pooled and by country

Model	Pooled	Haiti	Kenya	Nigeria	Uganda	Ukraine	Zimbabwe
Overall	**0.32 (0.28, 0.36)**	**0.25 (0.20, 0.32)**	**0.25 (0.15, 0.39)**	0.61 (0.00, 2.84)	**0.40 (0.22, 0.61)**	**0.37 (0.30, 0.45)**	**0.40 (0.23, 0.63)**
I^2	*47% (0%, 79%)*						
Age[1]		*p=0.10*	***p<0.0001****	-	*p=0.90*	*p=0.85*	*p=0.92*
<15 years	0.05 (0.01, 0.17)	0.15 (0.04, 0.36)	**0.00 (0.00, 0.00)**	-	1.91 (0.00, 5.24)	1.54 (0.00, 8.76)	1.30 (0.00, 6.30)
15-44 years	0.30 (0.26, 0.35)	0.24 (0.19, 0.30)	**0.22 (0.12, 0.37)**	-	0.38 (0.17, 0.61)	0.36 (0.28, 0.44)	0.42 (0.21, 0.72)
>44 years	0.40 (0.30, 0.52)	0.40 (0.26, 0.62)	**0.38 (0.12, 1.13)**	-	0.22 (0.00, 0.77)	0.40 (0.26, 0.56)	0.25 (0.04, 0.61)
Sex		*p=0.32*	*p=0.75*	*p=1.0*	*p=0.84*	*p=0.029*	*p=0.97*
Female	0.36 (0.29, 0.43)	0.28 (0.20, 0.38)	0.23 (0.12, 0.43)	1.98 (0.00, >10)	0.40 (0.15, 0.72)	0.50 (0.35, 0.67)	0.41 (0.13, 0.83)
Male	0.29 (0.25, 0.35)	0.23 (0.17, 0.31)	0.27 (0.12, 0.51)	0.19 (0.00, 1.26)	0.46 (0.15, 0.81)	0.32 (0.25, 0.40)	0.42 (0.20, 0.72)
Entry Year[1,2]		*p=0.86*	*p=1.0*	-	*p=1.0*	*p=0.15*	*p=1.0*
2018	0.33 (0.25, 0.42)	0.27 (0.19, 0.38)	0.25 (0.07, 0.72)	-	0.23 (0.00, 0.80)	0.41 (0.28, 0.56)	0.02 (0.00, 0.20)
2019	0.38 (0.30, 0.48)	0.23 (0.15, 0.37)	0.27 (0.12, 0.60)	-	0.44 (0.14, 0.83)	0.50 (0.36, 0.66)	1.02 (0.00, 2.82)
2020	0.35 (0.27, 0.46)	0.30 (0.19, 0.47)	0.25 (0.09, 0.52)	-	0.31 (0.05, 0.69)	0.41 (0.29, 0.57)	0.25 (0.03, 0.66)
2021	0.26 (0.17, 0.38)	0.25 (0.15, 0.43)	0.12 (0.00, 0.46)	-	0.56 (0.06, 1.37)	0.17 (0.06, 0.35)	0.37 (0.14, 0.72)
2022	0.17 (0.06, 0.52)	-	-	-	0.50 (0.00, 1.83)	-	0.17 (0.04, 0.42)
Clinical Stage[1,3,4]		*p=0.20*	*p=0.69*	-	***p<0.0001****	-	*p=0.34*
Stage 1	0.50 (0.34, 0.73)	0.58 (0.28, 0.98)	0.38 (0.15, 0.99)	-	**0.46 (0.18, 0.77)**	-	0.54 (0.18, 1.18)
Stage 2	0.36 (0.26, 0.51)	0.32 (0.15, 0.32)	0.23 (0.10, 0.49)	-	**0.71 (0.16, 1.54)**	-	1.50 (0.44, 3.35)
Stage 3	0.14 (0.10, 0.20)	0.29 (0.22, 0.49)	0.23 (0.09, 0.48)	-	**0.00 (0.00, 0.00)**	-	0.72 (0.21, 1.50)
Stage 4	0.08 (0.06, 0.12)	0.19 (0.19, 0.43)	-	-	**0.00 (0.00, 0.00)**	-	-
ART Regimen[1,5]		*p=0.35*	*p=0.67*	-	*p=0.81*	*p=0.015*	*p=0.86*
DTG	0.30 (0.25, 0.36)	0.26 (0.20, 0.33)	0.25 (0.14, 0.42)	-	0.29 (0.07, 0.63)	0.37 (0.28, 0.49)	0.33 (0.16, 0.55)
EFV	0.38 (0.30, 0.49)	0.43 (0.20, 0.93)	0.18 (0.05, 0.59)	-	0.37 (0.09, 0.74)	0.39 (0.29, 0.50)	1.19 (0.08, 2.88)
Other	0.44 (0.27, 0.71)	0.11 (0.07, 0.35)	-	-	0.77 (0.19, 1.62)	1.00 (0.47, 1.68)	0.24 (0.00, 0.74)

[1]Insufficient cases to estimate by subgroup for Nigeria.
[2]2022 data were not extracted for Haiti, Kenya, Nigeria, or Ukraine.
[3]Not reported in Ukraine data.
[4]Insufficient cases to estimate Stage 4 subgroup for Kenya and Zimbabwe.
[5]Insufficient cases to estimate Other subgroup for Kenya.
*Statistically significant at p<0.01.
ART: antiretroviral therapy; TB: tuberculosis; TPT: tuberculosis preventive treatment; DTG: dolutegravir; EFV: efavirenz

Supplemental Table S13. Adjusted hazard ratios for all-cause mortality comparing TPT initiation within 2 weeks of enrollment into care to never initiating TPT, pooled and by country

Model	Pooled	Haiti	Kenya	Nigeria	Uganda	Ukraine	Zimbabwe
Overall	**0.52 (0.49, 0.56)**	**0.44 (0.40, 0.49)**	**0.42 (0.31, 0.58)**	0.93 (0.32, 2.34)	**0.20 (0.13, 0.28)**	**0.63 (0.57, 0.69)**	**0.58 (0.45, 0.82)**
I^2	*90% (81%, 95%)*						
Age[1]		*p=0.68*	*p=0.87*	-	*p=0.98*	***p<0.0001****	*p=0.83*
<15 years	0.08 (0.05, 0.12)	0.42 (0.26, 0.65)	2.20 (0.00, 10.26)	-	0.24 (0.00, 0.86)	**0.00 (0.00, 0.00)**	1.39 (0.09, 6.22)
15-44 years	0.50 (0.45, 0.54)	0.44 (0.38, 0.50)	0.42 (0.28, 0.60)	-	0.22 (0.13, 0.32)	**0.60 (0.53, 0.68)**	0.55 (0.40, 0.85)
>44 years	0.60 (0.54, 0.68)	0.48 (0.41, 0.61)	0.36 (0.19, 0.65)	-	0.17 (0.04, 0.34)	**0.70 (0.60, 0.81)**	0.65 (0.36, 1.04)
Sex		*p=0.34*	*p=0.37*	*p=0.92*	*p=0.65*	*p=0.25*	*p=0.63*
Female	0.54 (0.48, 0.59)	0.46 (0.40, 0.54)	0.47 (0.30, 0.71)	1.04 (0.26, 3.54)	0.23 (0.12, 0.35)	0.68 (0.58, 0.80)	0.64 (0.38, 1.02)
Male	0.51 (0.46, 0.55)	0.42 (0.36, 0.50)	0.35 (0.21, 0.54)	0.69 (0.00, 6.84)	0.19 (0.08, 0.30)	0.60 (0.53, 0.68)	0.55 (0.38, 0.88)
Entry Year[1,2]		*p=0.033*	*p=0.60*	-	*p=0.35*	***p<0.0001****	*p=0.93*
2018	0.65 (0.56, 0.75)	0.54 (0.44, 0.65)	0.35 (0.13, 0.72)	-	0.14 (0.00, 0.34)	**0.95 (0.74, 1.22)**	0.00 (0.00, 0.27)
2019	0.56 (0.49, 0.64)	0.40 (0.33, 0.49)	0.30 (0.14, 0.55)	-	0.07 (0.02, 0.15)	**0.81 (0.66, 0.96)**	0.70 (0.00, 1.68)
2020	0.54 (0.47, 0.61)	0.49 (0.40, 0.65)	0.52 (0.30, 0.92)	-	0.15 (0.03, 0.30)	**0.57 (0.48, 0.66)**	0.51 (0.22, 0.93)
2021	0.37 (0.32, 0.43)	0.35 (0.26, 0.47)	0.32 (0.10, 0.67)	-	0.96 (0.44, 1.68)	**0.36 (0.30, 0.44)**	0.34 (0.18, 0.61)
2022	0.42 (0.24, 0.74)	-	-	-	0.57 (0.09, 1.24)	-	0.42 (0.26, 0.81)
Clinical Stage[1,3]		***p=0.0027***	***p<0.0001****	-	*p=0.90*	-	*p=0.44*
Stage 1	0.57 (0.47, 0.68)	**0.60 (0.48, 0.76)**	**0.83 (0.38, 2.02)**	-	0.18 (0.09, 0.29)	-	0.99 (0.57, 1.82)
Stage 2	0.42 (0.34, 0.51)	**0.37 (0.33, 0.54)**	**0.52 (0.32, 0.88)**	-	0.34 (0.11, 0.66)	-	0.68 (0.33, 1.23)
Stage 3	0.57 (0.47, 0.70)	**0.62 (0.30, 0.47)**	**0.33 (0.16, 0.55)**	-	0.33 (0.00, 0.86)	-	0.39 (0.12, 0.80)
Stage 4	0.30 (0.24, 0.38)	**0.58 (0.50, 0.78)**	**0.00 (0.00, 0.00)**	-	0.31 (0.00, 1.14)	-	0.69 (0.00, 3.19)
ART Regimen[1]		***p=0.0089***	*p=0.59*	-	*p=0.73*	*p=0.018*	*p=0.48*
DTG	0.52 (0.48, 0.57)	**0.43 (0.38, 0.51)**	0.42 (0.27, 0.63)	-	0.49 (0.27, 0.73)	0.58 (0.51, 0.66)	0.75 (0.53, 1.31)
EFV	0.63 (0.55, 0.72)	**0.66 (0.54, 0.83)**	0.28 (0.15, 0.50)	-	0.07 (0.00, 0.15)	0.65 (0.55, 0.78)	0.81 (0.18, 1.77)
Other	0.61 (0.49, 0.77)	**0.47 (0.53, 0.96)**	0.32 (0.00, 1.71)	-	0.11 (0.00, 0.26)	1.02 (0.67, 1.45)	0.29 (0.11, 0.55)

[1]Insufficient cases to estimate by subgroup for Nigeria.

[2]2022 data were not extracted for Haiti, Kenya, Nigeria, or Ukraine.

[3]Not reported in Ukraine data.

*Statistically significant at p<0.01.

ART: antiretroviral therapy; TB: tuberculosis; TPT: tuberculosis preventive treatment; DTG: dolutegravir; EFV: efavirenz

Supplemental Figure S9. Adjusted hazard ratios of tuberculosis diagnosis comparing TPT initiation within 2 weeks of enrollment into care to never initiating TPT, pooled and by country

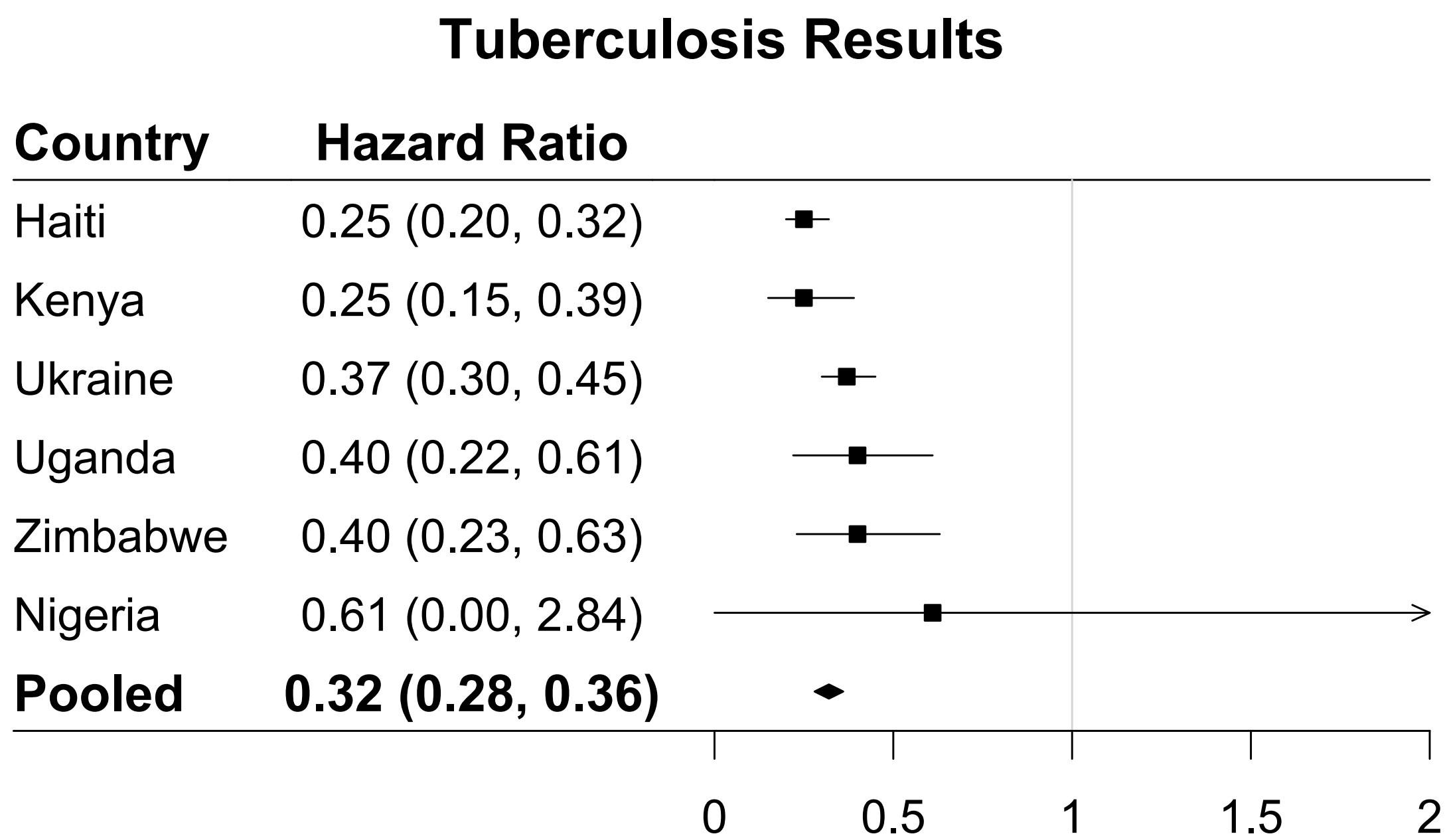

Supplemental Figure S10. Adjusted hazard ratios of all-cause mortality comparing TPT initiation within 2 weeks of enrollment into care to never initiating TPT, pooled and by country

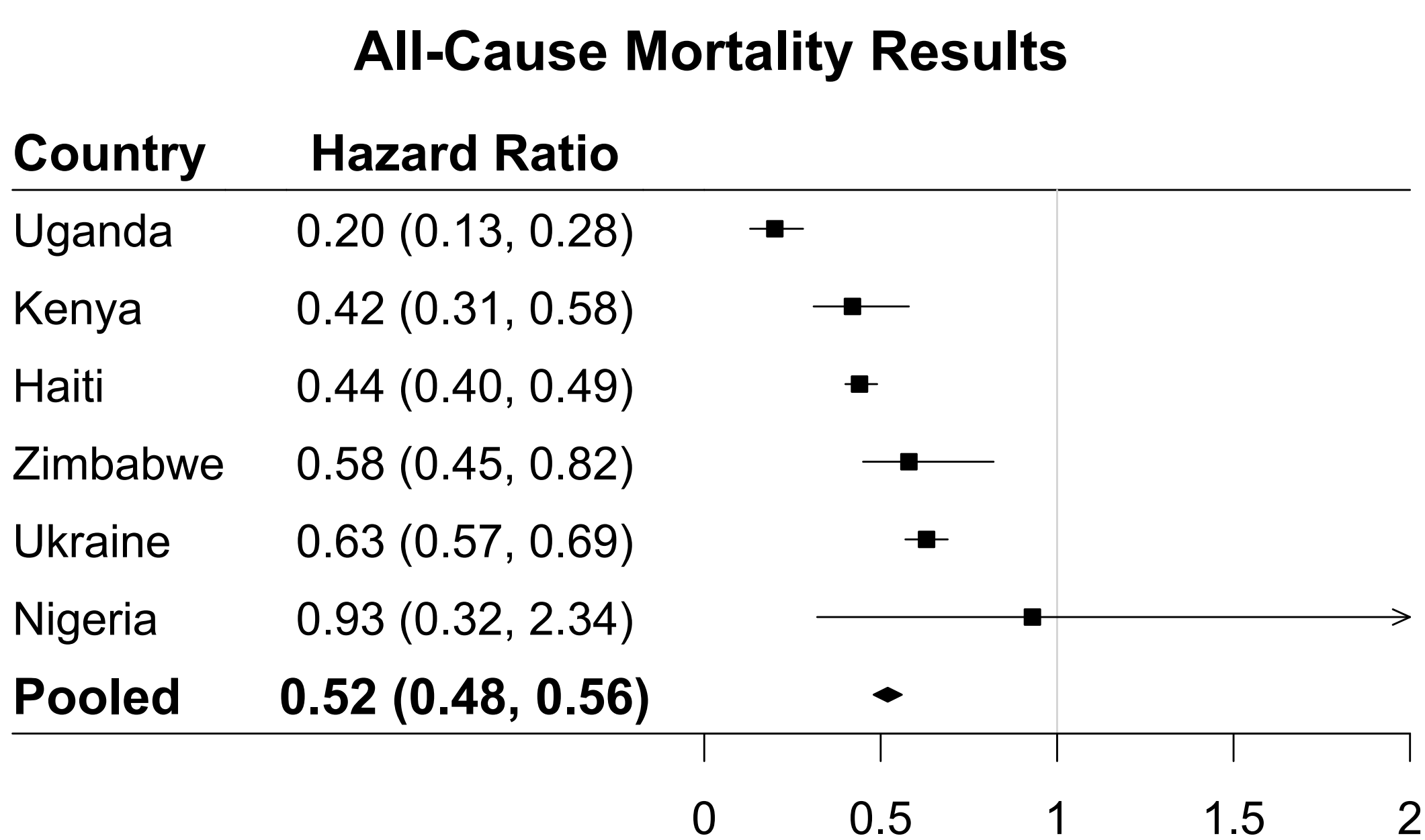

Supplemental Figure S11. Pooled adjusted hazard ratios of tuberculosis diagnosis comparing TPT initiation within 2 weeks of enrollment into care to never initiating TPT, overall and within population subgroups

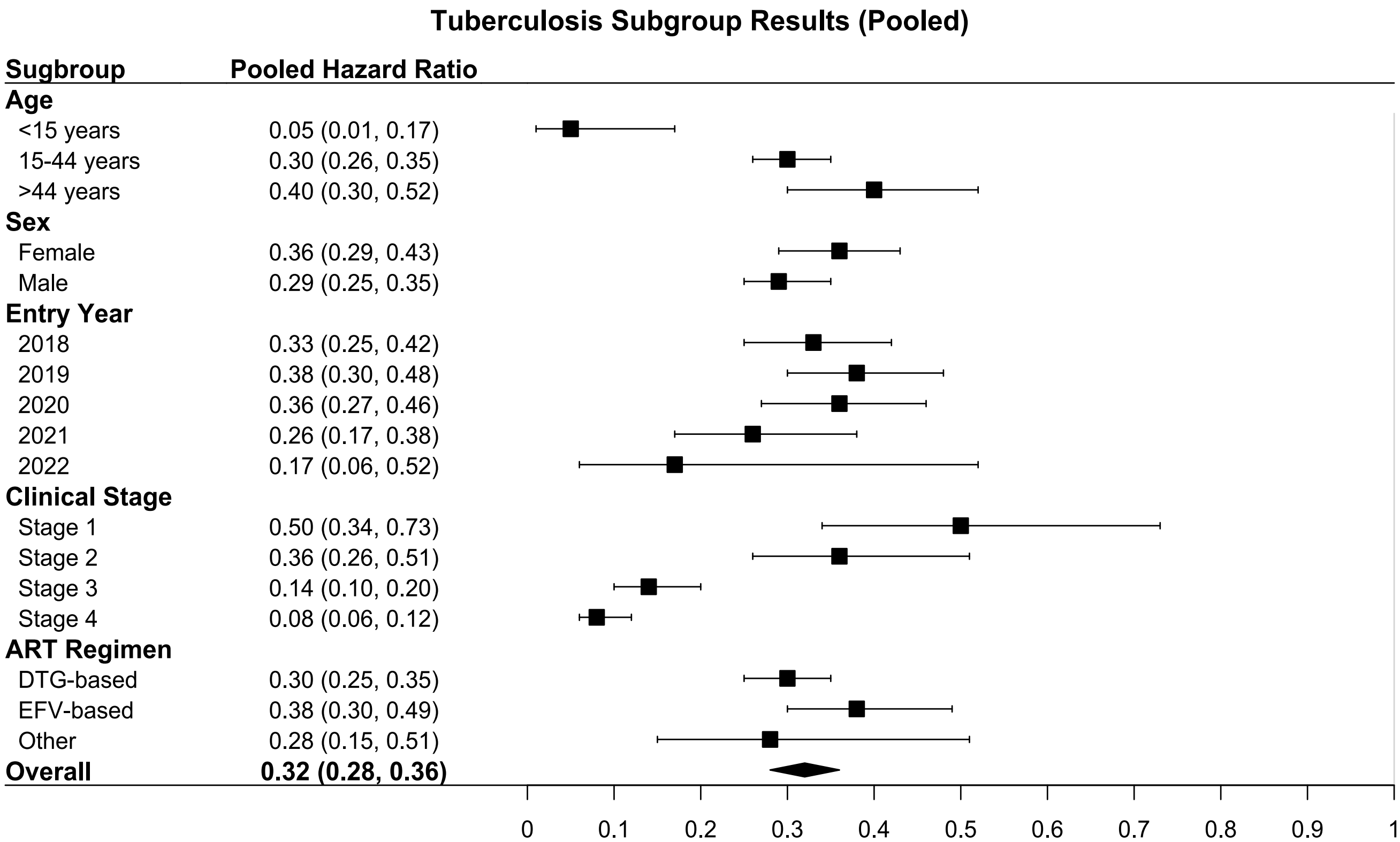

ART: antiretroviral therapy; TB: tuberculosis; TPT: tuberculosis preventive treatment; DTG: dolutegravir; EFV: efavirenz

Supplemental Figure S12. Pooled adjusted hazard ratios of all-cause mortality comparing TPT initiation within 2 weeks of enrollment into care to never initiating TPT, overall and within population subgroups

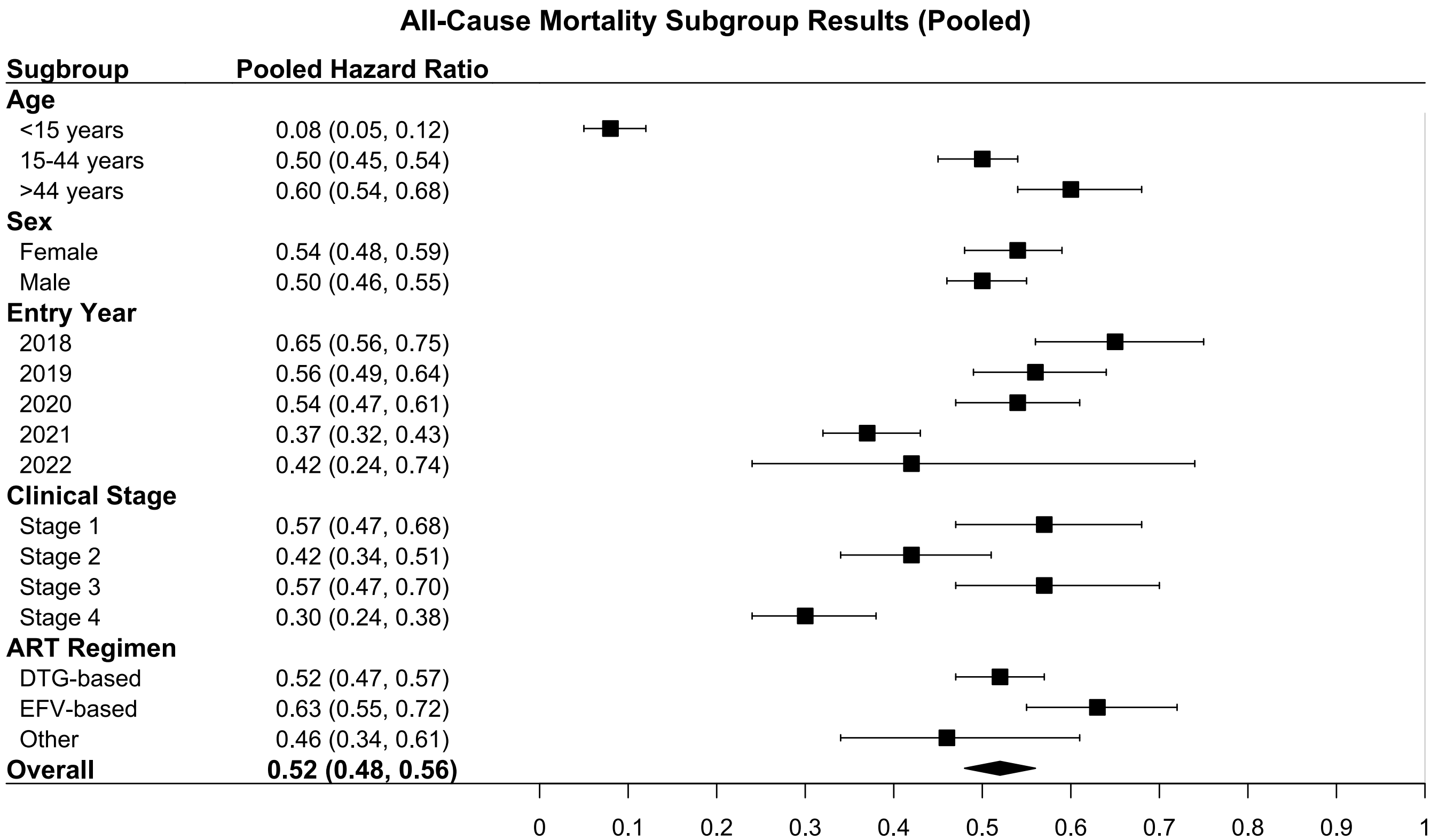

ART: antiretroviral therapy; TB: tuberculosis; TPT: tuberculosis preventive treatment; DTG: dolutegravir; EFV: efavirenz

REFERENCES

1. Joint United Nations Programme on HIV/AIDS. AIDS, crisis and the power to transform: UNAIDS Global AIDS Update 2025. Geneva: Joint United Nations Programme on HIV/AIDS, 2025.

2. World Health Organization. Global tuberculosis report 2025. Geneva: World Health Organization, 2025.

3. PEPFAR. 2024 Country operational plan guidance for all PEPFAR countries. Washington, D.C.: U.S. President's Emergency Plan for AIDS Relief, 2024.
